# From Industrial Waste to Multistage Applications: Ultralight Lignin‐Based Aerogel with Situ Vertically Oriented Structure for Photothermal‐Assisted Pb^2+^ Adsorption in Wastewater and Reuse as Efficient Output and Stability Triboelectric Materials

**DOI:** 10.1002/advs.202513337

**Published:** 2025-09-30

**Authors:** Boyu Du, Yuxin Yang, Lingjing Huang, Yuxuan Xie, Xing Wang, Jinghui Zhou, Xingxiang Ji, Lupeng Shao

**Affiliations:** ^1^ Key Laboratory of Pulp and Paper Science & Technology of Ministry of Education Qilu University of Technology (Shandong Academy of Sciences) Jinan 250353 P. R. China; ^2^ Guangxi Key Laboratory of Clean Pulp & Papermaking and Pollution Control School of Light Industry and Food Engineering Guangxi University Nanning 530004 P. R. China; ^3^ Liaoning Key Lab of Lignocellulose Chemistry and Bio Materials Liaoning Collaborative Innovation Center for Lignocellulosic Biorefinery College of Light Industry and Chemical Engineering Dalian Polytechnic University Dalian 116034 P. R. China

**Keywords:** directional layered structure, lignin‐based aerogel, photothermal‐assisted adsorption, sulfonated lignin, triboelectric nanogenerator

## Abstract

In this work, the ultralight lignin‐based aerogel (GSPCAA) with situ vertically oriented structure is developed through a simple and green synthesis strategy for efficient photothermally assisted adsorption of Pb^2+^, and then reused as a lignin‐based triboelectric nanogenerator (TENG). Sulfonation is first performed on lignin's structure to enhance its water solubility and introduce the number of active adsorption sites. Then, GSPCAA is prepared using directed freezing and ionic crosslinking methods. The synergistic interactions between sulfonated lignin (SL) and graphene (GO) components endow GSPCAA with excellent photothermal properties, thereby generating the localized thermal environment on GSPCAA's surface to further improve the adsorption capacity of Pb^2+^. Finally, the spent adsorbent (GSPCAA‐Pb^2+^) is applied as lignin‐based TENG for the first time to achieve multi‐stage application of lignin‐based adsorbent. It displays excellent electric signal output performances. Meanwhile, a 3 × 3 self‐powered array is designed to accurately identify and visualize the pressure distributions of different load objects. Besides, combined with deep learning model algorithms, an overall recognition accuracy of 98.5% is achieved in the classification and identification of 11 different objects, fully indicating its application potential in the field of smart homes.

## Graphical abstract

Ultralight lignin‐based aerogel with situ vertically oriented structure for photothermally assisted adsorption of Pb^2+^, and then reused as lignin‐based triboelectric materials.

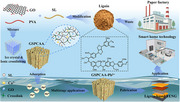

## 1 Introduction

With heavy industry's accelerated growth in recent years, water pollution problems caused by heavy metals, due to untreated or improper treatment are gradually attracting widespread attention from mankind.^[^ ^1^, ^2^ ^]^ At present, the primary methods for removing heavy metal ions include chemical precipitation, electrodialysis, ion exchange, and membrane separation et al.^[^ ^3^, ^4^, ^5^, ^6^ ^]^ However, these methods have inherent drawbacks in practical applications, such as high production costs and energy consumption, as well as the generation of by‐products like sludge from excessive chemical additives, which can cause secondary pollution. Therefore, developing more efficient and cost‐effective methods for heavy metal ion removal is very imperative. Adsorption has been reported as a promising approach for heavy metal ion removal due to its simplicity, cost‐effectiveness, high efficiency, and minimal secondary pollution.^[^ ^7^, ^8^, ^9^, ^10^, ^11^ ^]^ Commonly used adsorbents in practical applications include activated carbon, carbon nanotubes (CNTs), functionalized magnetic nanoparticles, synthetic polymers, and biomass‐based materials.^[^ ^8^, ^12^, ^13^, ^14^ ^]^ Thus, it is particularly important to prepare environmentally friendly adsorption materials with high adsorption capacity.

Lignin is the second most abundant natural renewable raw material after cellulose, with the structure primarily composed of three distinct phenylpropane structural units: syringyl (S), guaiacyl (G), and *p*‐hydroxyphenyl (H).^[^ ^15^, ^16^ ^]^ Currently, over 50 million tons of industrial lignin are produced as a by‐product worldwide, but only 2%–10% of it is effectively utilized to produce high‐value materials and chemicals.^[^ ^17^, ^18^ ^]^ Lignin typically possesses various functional groups, including carboxyl (‐COOH), hydroxyl (─OH) and methoxy groups (─OCH_3_), making it an ideal feedstock for the production of low‐cost and eco‐friendly adsorbents.^[^ ^19^ ^]^ For example, Ma et al. fabricated lignin‐based acrylic acid nanocomposites with the maximum adsorption capacity (Q_max_) of Pb^2^⁺ of 1.08 mmol g^−1^.^[^ ^20^ ^]^ Krapishevsky et al. synthesized a functionalized silica/lignin composite adsorbent, achieving the Q_max_ of Ni^2^⁺ of 77.11 mg g^−1^.^[^ ^21^ ^]^ However, in these published studies, unmodified lignin exhibited limited adsorption capacity due to its restricted number of active functional groups. Therefore, before preparing lignin‐based adsorbent, chemical modification of the structure of lignin needs to be performed, thereby significantly improving the number of adsorption active sites and ultimately enhancing its adsorption performances on heavy metal ions. Furthermore, most of lignin‐based adsorbents currently suffer from low specific surface area and limited spatial structure, which severely hinders the diffusion and mass transfer of heavy metal ions within the adsorbent matrix, resulting in limited capacity and sluggish adsorption kinetics.^[^ ^22^, ^23^, ^24^, ^25^ ^]^ It has been reported that adsorbents exhibiting outstanding photothermal conversion can create localized heating through solar energy absorption, thereby accelerating the thermodynamics of the adsorption process and significantly enhancing the adsorption performances of the material.^[^ ^25^, ^26^, ^27^, ^28^ ^]^ Notably, lignin displays good photothermal conversion capability due to *π‐π* interactions, enabling its effective application as an eco‐friendly photothermal agent in intelligent material fields.^[^ ^29^, ^30^ ^]^ Based on these factors, we hypothesize that developing novel lignin‐based adsorbents with superior photothermal performances can further enhance its adsorption capacity for heavy metal ions.

One of the key challenges in adsorption technology is the post‐use disposal of the spent adsorbents. At present, the spent adsorbent is generally discarded as waste, which significantly undermines the environmental sustainability of the adsorption process and pushes up the costs of application. Hence, reutilizing spent adsorbents represents the optimal strategy to maximize both economic and environmental benefits. For instance, Li et al. investigated the adsorption of phosphorus by magnetic lignin nanoparticles and further utilized the phosphorus‐saturated adsorbents as renewable slow‐release complex fertilizers.^[^ ^31^ ^]^ Ren et al. proposed the multilevel application strategy for lignin‐based adsorbents, converting Cu^2^⁺‐loaded spent adsorbents into CuO/C catalysts for efficient lignin hydrogenolysis after heavy metal removal.^[^ ^32^ ^]^ Considering the scarcity and significance of these studies, producing high‐performance adsorbents from green sustainable materials and further valorizing spent adsorbents through continuous high‐value applications holds great importance.

Nowadays, the coupling between the triboelectric effect and electrostatic induction is utilized, triboelectric nanogenerator (TENG) serves as a sustainable and green power supply for flexible electronics through the collection of low‐frequency and distributed mechanical energy.^[^ ^33^, ^34^ ^]^ The primary elements of TENG are the triboelectric electrodes that possess varying abilities to capture and donate electrons.^[^ ^35^, ^36^ ^]^ Under the concept of “returning to nature,” the use of renewable and eco‐friendly bio‐polymers as advanced triboelectric electrodes for building high‐performance biomass‐based TENG has drawn increasing attention in recent years.^[^ ^36^, ^37^ ^]^ As of now, lignin has been recognized as an effective anode material for enhancing triboelectric charge density in biomass‐based TENG.^[^ ^38^ ^]^ On one hand, the structure of lignin endows biomass‐based TENG with excellent mechanical strength and wear resistance, maintaining structural stability during the friction and enabling long‐term stable operation.^[^ ^39^ ^]^ On the other hand, lignin's phenolic hydroxyl groups and other active functional groups can generate significant contact electrification effects with other materials during the contact‐separation processes.^[^ ^40^ ^]^ Unfortunately, the optimal electrical output performances of most of lignin‐based TENGs remain limited. In theory, through the hybridization between *π* orbitals of lignin and d orbitals of transition metals, the departure of electrons from the domains in the *π*‐d system adjusts their charged states, leading to the expansion of the electron cloud and the enhancement of the polarity of the material.^[^ ^41^ ^]^ It can promote triboelectric charge generation in the lignin‐based TENG.^[^ ^41^ ^]^ Such as, Du et al. proposed the utilization of transition metal (Fe) conjugated coordination lignin as the tribopositive nanofiller in boosting the triboelectric behaviors of green and recyclable CMC‐based TENG.^[^ ^42^ ^]^ Based on this strategy, it is predicted that GSPCAA after Pb^2^⁺ adsorption may induce spatial charge redistribution in lignin, thereby enhancing the lignin‐based TENG's surface charge density and triboelectric polarity. In short, these advantageous properties may endow the spent adsorbents after heavy metal ion adsorption with significant potential for constructing lignin‐based TENG with high performance.

**Figure 1:**
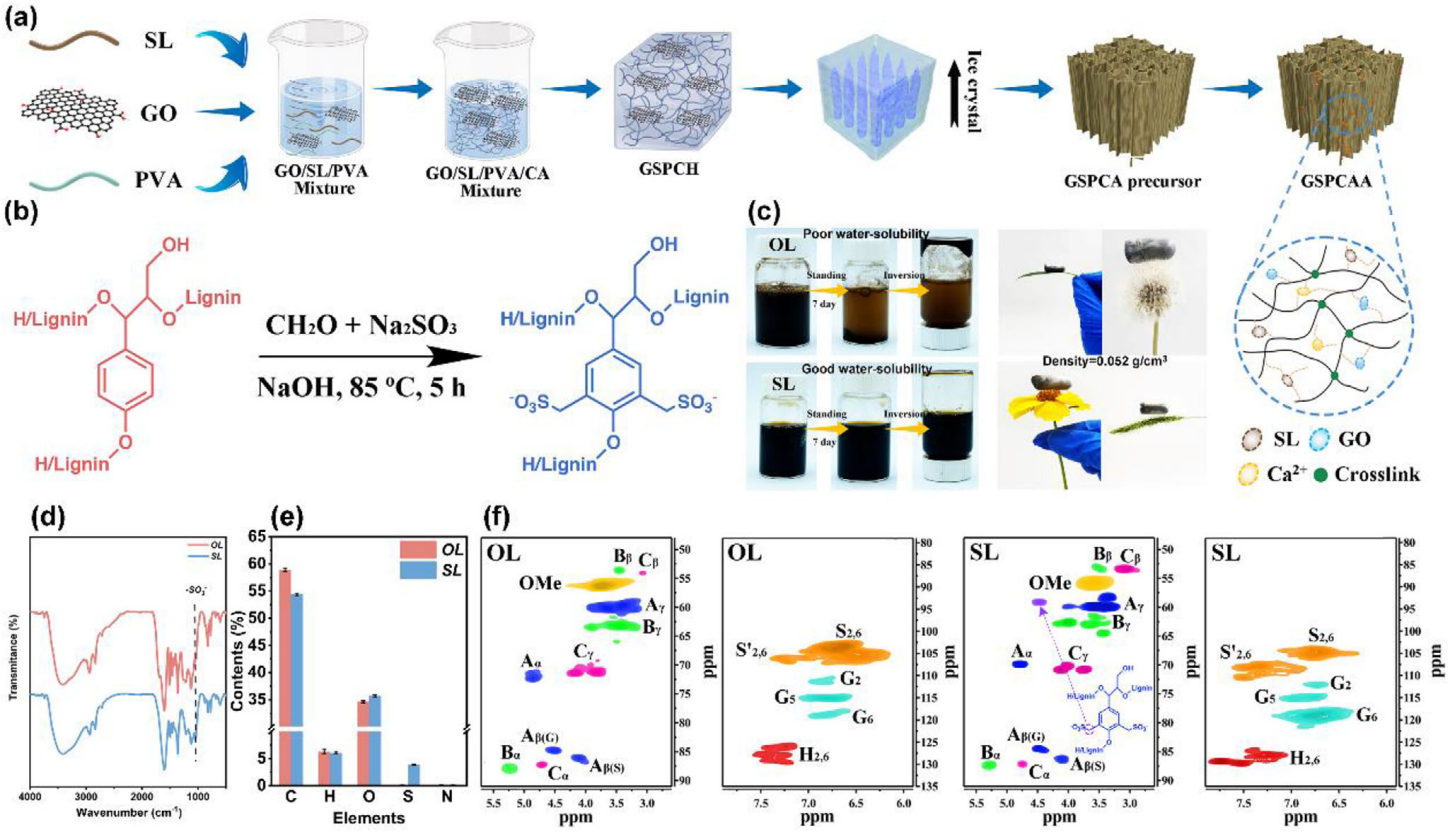
Design of GSPCAA and characterization of lignin. a) Schematic showing the synthetic steps and photos showing ultralight property, b) the synthesis scheme of SL, c) digital photographs for water solubility test of OL and SL, d) FT‐IR spectra of OL and SL, e) the contents of C, H, O, S, N elements of OL and SL, and f) 2D‐HSQC of OL and SL.

In this work, a novel ultralight lignin‐based aerogel (GSPCAA) was developed for efficient photothermal‐assisted adsorption of Pb^2+^ from wastewater (**Figure** **1**a), and the density was only 0.052 g cm^−3^. Original lignin (OL) was first subjected to sulfonation modification. By introducing abundant sulfonic acid groups into the OL structure, excellent water solubility and numerous adsorption active sites of sulfonated lignin (SL) were generated. Subsequently, high‐strength GSPCAA with situ vertically oriented structure was fabricated via directional freezing and ionic crosslinking methods. In the construction of GSPCAA, SL provided abundant active sites for Pb^2+^ adsorption. At the same time, graphene (GO) as a reinforcing phase effectively prevented undesirable volume shrinkage and layered structure stacking during the ionic crosslinking, thereby causing enhance exposure of adsorption active sites. Significantly, GSPCAA achieved excellent photothermal response via SL‐GO component synergy, generating localized thermal islands on its surface to accelerate the thermodynamics of Pb^2+^ adsorption. Based on these advantages, GSPCAA exhibited remarkable Pb^2+^ adsorption capacity under the light irradiation, with the Q_max_ of 315.8 mg g^−1^, demonstrating significant practical application potential. Finally, considering the recycling of the spent adsorbents, Pb^2^⁺‐adsorbed GSPCAA (GSPCAA‐Pb^2+^) was utilized to fabricate lignin‐based TENG, enabling multi‐stage applications of lignin‐based adsorbents. Meanwhile, a self‐powered 3 × 3 array was designed for precise identification and real‐time visualization of pressure distribution from different loaded objects. In addition, through integrating deep learning model algorithms, an overall recognition accuracy of 98.5% was also achieved in classifying and identifying 11 different objects.

## 2 Results and Discussion

### 2.1 Sulfonation of Lignin

The synthesis of SL is illustrated in Figure 1b. In this work, formaldehyde (HCHO) and sodium sulfite (Na_2_SO_3_) provided the methyl group and the sulfonated group (SO_3_^−^) for sulfonation, respectively. This reaction proceeded via electrophilic substitution. Under the alkali condition, phenolic groups in lignin were converted to active electrophiles, and sodium sulfonate methyl derivative was formed by the nucleophilic addition of the sodium sulfite anion.^[^ ^43^, ^44^ ^]^ Figure 1c exhibits the water solubilities of OL and SL. In detail, 0.2 g of lignin was dissolved in 5 mL of deionized water and stored for 7 days. Apparently, SL displayed better water solubility than OL, which allowed SL to be fully incorporated into GSPCAA during the subsequent preparation process. Then, various technologies were performed to verify the success of lignin sulfonation. Figure 1d shows the Fourier transform infrared spectroscopy (FT‐IR) spectra of OL and SL. The signal at 3422 cm^−1^ could be attributed to ─OH stretching in phenolic and aliphatic structures, and the signals at 2835 and 2966 cm^−1^ were explained to the C─H stretching vibration of CH_3_ and CH_2_ in aliphatic side chains, respectively.^[^ ^44^ ^]^ As compared with OL, the benzene ring basic structure (1601, 1515, and 1443 cm^−1^) of OL remained in SL, suggesting that the aromatic structure of lignin was not destroyed during the sulfonation process. In particular, a new vibrational peak representing the O═S═O bond at 1040 cm^−1^ was observed in SL, which revealed the successful introduction of SO_3_^−^ group in lignin.^[^ ^44^ ^]^ After the sulfonation process, the content of sulfur (S) increased significantly from 0.09% (OL) to 3.82% (SL), which indicated the SO_3_^−^ group was successfully grafted onto the structure of OL (Figure 1e). Lignin was also characterized by 2D‐heteronuclear single quantum coherence (2D‐HSQC). As shown in Figure 1f, no significant changes of signals were found in the structure of lignin before and after the sulfonation process, confirming that the modification process did not change lignin's core framework. Notably, as compared with OL, a new signal at δC/δH = 59.8/4.5 ppm was appeared in the side‐chain region of SL, which could be interpreted as the signal of ─CH_2_SO_3_^−^ group.^[^ ^44^ ^]^ Finally, Zeta potential of OL and SL at various pH values are demonstrated in Figure S1 (Supporting Information). It was observed that the negative zeta potential of both OL and SL was significantly strengthened as the pH value increased, owing to deprotonation.^[^ ^45^, ^46^ ^]^ Meanwhile, the negative zeta potential of SL was more prominent than that of OL, which might be related to the high content of ─SO_3_^−^ group in SL.^[^ ^47^, ^48^ ^]^ Based on the above results, we confirmed that SL had been successfully prepared.

### 2.2 Characterization of GSPCAA

**Figure 2:**
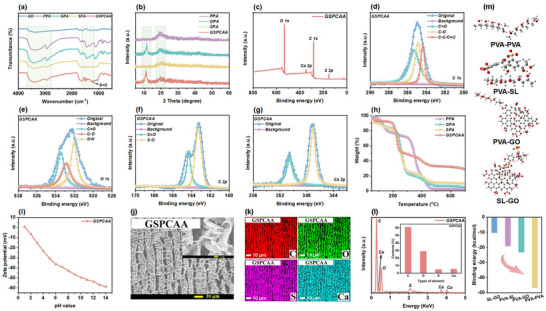
Characterizations of GSPCAA. a) FT‐IR spectra of GO, PPA, GPA, SPA and GSPCAA, b) XRD patterns of PPA, GPA, SPA and GSPCAA, c) XPS survey spectra of GSPCAA, d–g) high‐resolution C 1s spectra, O 1s spectra, S 2p spectra and Ca 2p spectra of GSPCAA, (h) TGA curves of PA, GPA, SPA and GSPCAA, i) zeta potential of GSPCAA, j) SEM images of GSPCAA, k,l) EDS elemental mappings of GSPCAA, and m) the calculated binding energy values of the four pairs of components in the networks of GSPCAA based on the density functional theory (DFT) optimized structures.

Following the sulfonation of lignin, GSPCAA with a multiscale structure was fabricated by situ vertical orientation combined with the mild ionic crosslinking process. **Figure** **2**a shows the FT‐IR spectra of GO, pure PVA aerogel (PPA, without GO and SL), GO/PVA aerogel (GPA, without SL), SL/PVA aerogel (SPA, without GO), and GSPCAA. GO had the characteristic peaks at 1717 and 3388 cm^−1^, which could be attributed to the stretching vibrations of C═O and ─OH groups, respectively.^[^ ^49^ ^]^ The strong absorption peaks at 3420 and 915 cm^−1^ of PPA were interpreted as the ─OH tensile vibration and the bending vibration of CH_2_, respectively.^[^ ^50^, ^51^ ^]^ Meanwhile, the signal intensities of ─OH and ─COOH in GSPCAA were weaker than those of other samples, suggesting the possible presence of the ionic coordination bonds obtained via Ca^2+^ cross‐linking.^[^ ^52^ ^]^ In addition, the stretching vibrational peaks of C═O and S═O in GSPCAA changed to 1615 and 1261 cm^−1^, respectively, which revealed the interaction between these functional groups and Ca.^2+[^ ^52^ ^]^

XRD patterns of all samples are given in Figure 2b and Figure S2 (Supporting Information). It was noticed that PPA exhibited the distinct diffraction peak at 19.8°, which could be attributed to the square lattice structural plane of (101).^[^ ^53^, ^54^ ^]^ At the same time, the peak of 19.8° was ascribed to the presence of strong intermolecular and intramolecular interactions. For GSPCAA, the diffraction peak was decreased with the addition of SL and GO, suggesting that the crystallinity of the cross‐linked PVA chain was altered. This phenomenon might be due to specific interactions between SL and GO, and PVA groups in the presence of Ca^2+^.^[^ ^52^ ^]^ The chemical compositions and bonding structure of GSPCAA were investigated through XPS technology. As shown in Figure 2c, the binding energies at 285.8, 533.2, 164.6, and 351.4 eV were corresponded to the diffraction peaks of C, O, S, and Ca elements, respectively. This result demonstrated that GSPCAA might be carried a giant quantity of sulfur‐containing and oxygen‐containing functional groups, which were more favorable for the adsorption of Pb^2+^. In detail, Figure 2d displays the C 1s spectrum, which was fitted to three peaks designated as C═O (285.58 eV), C─O (284.84 eV), and C─C/C═C (284.48 eV), respectively. For O 1s spectrum, three peaks were fitted and corresponded to C═O (533.77 eV), C─O (532.91 eV), and O─H (532.01 eV), respectively (Figure 2e). As could be found in Figure 2f, two peaks were exhibited in the S 2p spectrum, and the binding energies of 164.48 and 163.27 eV were S═O and S─O, respectively. In addition, two peaks at 350.88 and 347.08 eV were discovered for Ca 2p spectrum, respectively (Figure 2g). These results were consistent with the FT‐IR spectra, further confirming the successful cross‐linking on GSPCAA.

Afterward, we explored the thermal stability of different aerogels via TGA curves. Obviously, the first weight loss peak of all samples was the desorption of the bound water, which occurred at ≈200 °C (Figure 2h). Because PVA, SL, and GO contained a large number of hydrophilic groups, they were highly susceptible to absorb moisture from air.^[^ ^53^, ^54^ ^]^ The second weight loss peak occurred between 200 and 400 °C, which was mainly due to the decomposition of the unstable oxygen‐containing groups of the aerogel. Finally, the weight loss observed between 400 and 700 °C originated from the decomposition of the aerogel's more stable components until the weight was constant. The superior thermal stability of SL and GO combined, with the cross‐linked network formed with PVA, delayed the decomposition of GSPCAA. Therefore, the addition of SL and GO to PVA was conducive to the enhancement of the thermal stability of GSPCAA. The amount of charge of materials plays an important role in the adsorption of Pb^2+^. As shown in Figure 2i, Zeta potential of GSPCAA gradually decreased with increasing pH value, and the zero charge (pH_pzc_) was ≈1.2. This phenomenon indicated that the positively charged nature of the GSPCAA surface at low pH could be attributed to protonation of ─COOH, ─OH and ─SO_3_^−^ group.^[^ ^55^, ^56^ ^]^ As pH value continued to increase, an increase in the negative zeta potential of GSPCAA originated from the ionization of oxygen‐ and sulfur‐containing functional group.^[^ ^56^, ^57^ ^]^

The morphologies and structures of all samples were observed by scanning electron microscope (SEM). As shown in Figure S5 (Supporting Information) and Figure 2j, the structure of GO was a flat layered surface with a smooth surface and crumpled edge due to the interaction of the oxygen‐containing groups of GO.^[^ ^58^, ^59^ ^]^ Meanwhile, the parallel pore structure of PPA underwent significant degradation, manifesting as deformed and wrinkled morphology alongside structural collapse. By comparison, the layers structure of the GSPCAA appeared unwrinkled and non‐stacked, with its pore structure exhibiting superior mechanical integrity to other aerogels, indicating that SL and GO performed a good structural support role in the aerogel.^[^ ^52^ ^]^ The formation of hydrogen bonds and chemical cross‐links among the three components in GSPCAA was pivotal, as the resulting strong bonding overcame the capillary forces arising from ice crystal sublimation and the expansion stresses induced by ice crystal growth, thereby creating a highly aligned porous 3D network structure.^[^ ^52^ ^]^ In addition, disordered‐GSPCAA (D‐GSPCAA) displayed the disordered porous structure during the non‐oriented freezing process, suggesting that the oriented freeze‐casting process was the main reason for the formation of vertically oriented structures. Thus, GSPCAA with multiscale structure enabled in situ vertical orientation provided uniform oriented channels and excellent specific surface area, which might be more helpful for the mass transfer of Pb^2+^ during the adsorption process. What is more, the elemental mapping of GSPCAA is performed in Figure 2k,l, which are mainly composed of C, O, S, and Ca elements with the contents of 60.94%, 28.87%, 4.84% and 5.35%, respectively. Remarkably, the homogeneous distribution of S element was identical to that of other elements, indicating that SL was uniformly dispersed in GSPCAA.

To reveal the distinct roles of hydrogen bonds in network establishment, we employed density functional theory (DFT) calculations to determine the binding energies associated with four different types of hydrogen bonds in GSPCAA, and the results are depicted in Figure 2m. Obviously, the hydrogen bonds within the PVA chains were the strongest, highlighting the significance of the PVA networks as the structural foundation of the GSPCAA. In contrast, the hydrogen binding energies between PVA chains and SL, as well as GO, were relatively weak, and the difference among these binding energies was not significant. It indicated the involvement of both types of hydrogen bonds to a certain extent in the assembly of polymer networks and had a competitive relationship between them. The hydrogen binding energy between SL and GO was the lowest, which suggested that the interactions lacked stability and were susceptible to mechanical fracture.

**Figure 3:**
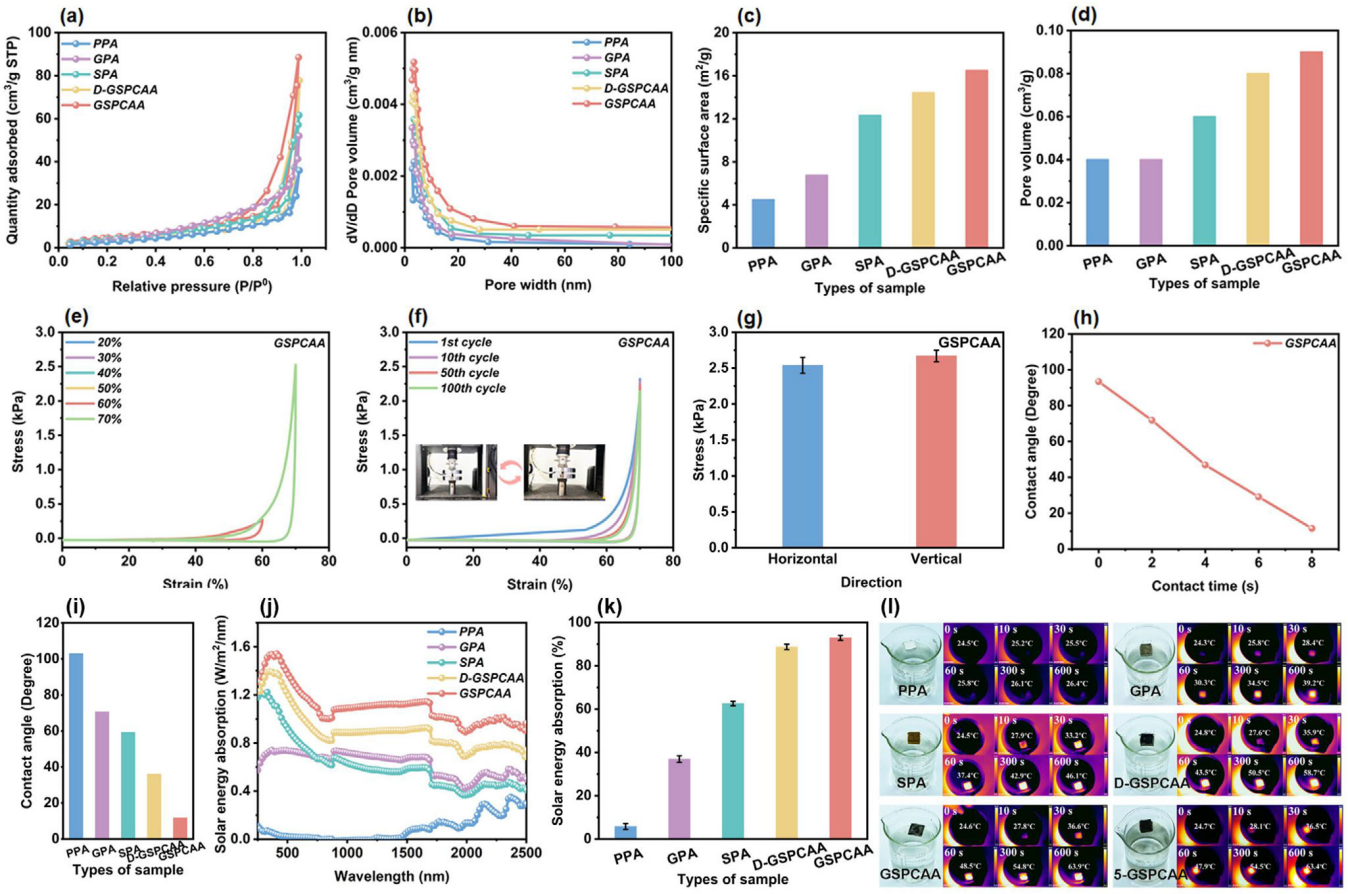
Characterization, mechanical property, hydrophilicity and photothermal performance of GSPCAA. a) N_2_ adsorption‐desorption isotherms and b) BJH pore size distribution of PPA, GPA, SPA, D‐GSPCAA and GSPCAA, c) BET surface area and d) pore volume results of PPA, GPA, SPA, D‐GSPCAA and GSPCAA, e) compressive stress vs strain curves of GSPCAA during loading unloading cycles with increasing strain amplitude, f) compressive stress vs strain curves of GSPCAA under different cycles with 70% compressive strain, g) compressive stress of GSPCAA under different direction with 70% compressive strain, h) the contact angle of water droplets falling on GSPCAA at different content time, i) the contact angle of water droplets falling on PPA, GPA, SPA, D‐GSPCAA and GSPCAA, j) solar energy absorption spectra and k) solar energy absorption efficiency of PPA, GPA, SPA, D‐GSPCAA and GSPCAA, and l) thermal infrared images of PPA, GPA, SPA, GSPCAA, D‐GSPCAA and 5‐GSPCAA in water under the light irradiation.

In addition, N_2_ adsorption‐desorption isotherm was further carried out. All curves appeared the type IV isotherm with a H4 hysteresis loop in a relatively high‐pressure range (**Figure** **3**a), suggesting the presence of mesopores/macropores.^[^ ^15^ ^]^ Meanwhile, all samples had no significant adsorption at relatively low pressure, indicating the absence of micropores (Figure 3b). Figure 3c,d display the detailed BET surface area and pore volume results. Clearly, GSPCAA had the largest BET specific area and the volume of pores than other aerogels, suggesting that the incorporation of SL and GO together with the directional freeze‐casting process contributed to improve the specific surface area and porosity of GSPCAA.^[^ ^52^ ^]^ On one hand, GO and SL could be used as reinforcing phases to improve the volume shrinkage and lamellar structure of GSPCAA during the cross‐linking process. On the other hand, the directional freeze‐casting process could form larger and denser vertically oriented lamellar structures, which exposed more active sites for the adsorption of Pb^2+^.

### 2.3 Mechanical Property and Hydrophilicity of GSPCAA

The compressive experiment was used to verify the mechanical strength of GSPCAA. Figure 3e exhibits the different compressive stress–strain curves of GSPCAA. It was important to note that GSPCAA presented the ideal compressive property, which could withstand 70% strain without failure. Notably, both GSPCAA and D‐GSPCAA demonstrated superior mechanical strength compared to GPA and SPA (Figure S6, Supporting Information, 70% strain). This further confirmed that incorporating SL and GO as reinforcing phases enhanced the stability of the lignin‐based aerogel, facilitating its application and recycling. It was also consistent with the results of SEM. Besides, GSPCA possessed an outstanding cycling performance. As shown in Figure 3f, no significant reduction in strength was found for aerogels after the 1st compression, all the way up to 100th cycle, indicating that the dense porous structure could effectively withstand repeated compression. After 100 cycles of the compression tests, GSPCAA still retained more than 88.84% of the initial stress. The underlying mechanism involved strong interactions among the components, encompassing hydrogen bonding, coordination bonds, and entanglement of molecular chains. More importantly, GSPCAA also displayed excellent elasticity in all directions (Figure 3g). Therefore, the remarkable mechanical performances of GSPCAA could effectively extend its service life in practical applications.

The hydrophilicity of all samples was analyzed by contact angle measurements. As shown in Figure 3h,i, when SL and GO were both added to the PVA matrix, the water contact angle value of GSPCAA was 10.4° (8 s), which was lower than that of PPA, GPA, SPA, and D‐GSPCAA. This result was mainly attributed to the abundance of the hydrophilic groups in SL and GO, which led to the decrease of the hydrophobicity of the surface of the aerogel.^[^ ^52^ ^]^ Simultaneously, the water dropped on the surface of GSPCAA also rapidly penetrated into the interior of the aerogel, which indicated that the porous structure and the active groups of GSPCAA could promote the absorption of liquid. In short, GSPCAA with excellent hydrophilicity might be more conducive to the diffusion of Pb^2^, thereby effectively promoting the adsorption capacity of GSPCAA.

### 2.4 Photothermal Performance of GSPCAA and Its Effect on Pb^2+^ Adsorption Capacity

To investigate sunlight's enhancement effect on the Pb^2^⁺ adsorption capacity of GSPCAA, ultraviolet‐visible–near infrared spectroscopy (UV–vis–NIR) spectra of all samples were initially characterized. As shown in Figure 3j,k, PPA was poor over the range from 200 to 2500 nm. As a comparison, D‐GSPCAA and GSPCAA with the addition of SL and GO exhibited excellent solar energy absorbances, which were primarily ascribed to the strong light absorption abilities of SL and GO (Figure S7, Supporting Information).^[^ ^60^, ^61^ ^]^ Especially, the solar energy absorption of GSPCAA (91.74%) was higher than that of D‐GSPCAA (90.31%) after directional freeze‐drying process, suggesting that the dense and ordered multiscale structure of the aerogel might be more favorable for energy propagation. Then, the photothermal properties of all samples were further measured under simulated solar irradiation (Figure 3l). The images captured by the infrared thermal camera presented that PPA dispersed in water exhibited a gradual surface temperature rise of 1.9 °C within 600 s (from 24.5 to 26.4 °C). On the contrary, under the same simulated solar irradiation, the light‐generated heat was all well localized on GPA and SPA, which indicated that SL and GO both could be efficiently restrained thermal energy diffusion into bulk water. In addition, the surface temperatures of D‐GSPCAA and GSPCAA were both rapidly increased to 58.7 and 63.9 °C after 600 s, respectively, confirming the synergistic effect of SL and GO components in improving the photothermal performances of samples. It was worth noting that the surface temperature of GSPCAA was higher than that of D‐GSPCAA, further suggested that the multiscale structure of lignin‐based aerogel had an effect on its photothermal performance. These results were also consistent with their solar absorptivity in UV–vis–NIR spectra. More importantly, GSPCAA maintained exceptional photothermal stability through five solar irradiation on/off cycles (5‐GSPCAA), confirming its capacity for efficient and repeatable light‐to‐thermal energy conversion. It suggested that GSPCAA had excellent photothermal conversion efficiency and successfully form the localized heat islands under the sunlight.

**Figure 4:**
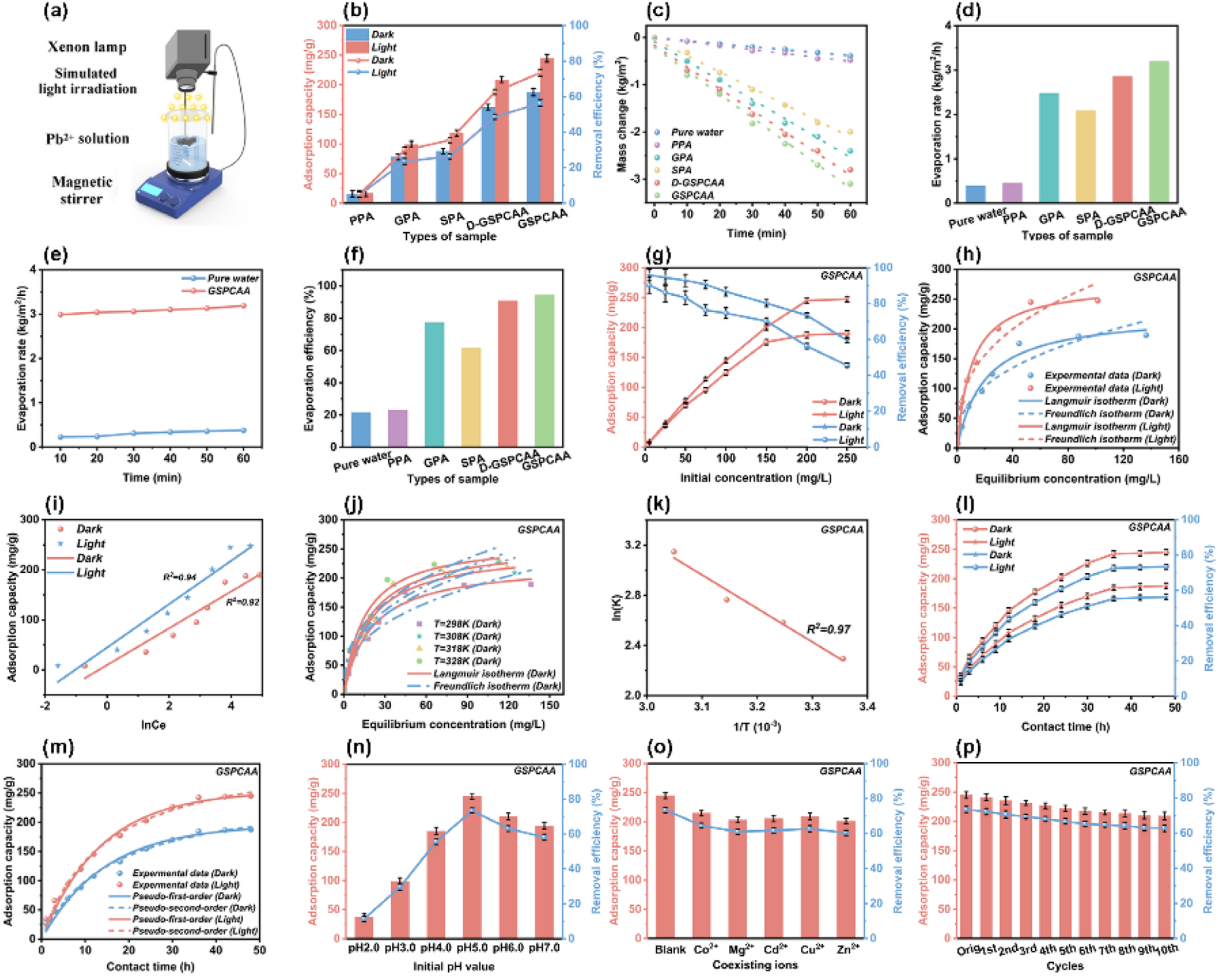
Photothermal and adsorption performances of GSPCAA. a) Schematic diagram for the adsorption of Pb^2+^ under the light irradiation, b) the effect of Pb^2+^ adsorption capacities of PPA, GPA, SPA, D‐GSPCAA and GSPCAA under the dark and light conditions, c) the water mass loss of pure water, PPA, GPA, SPA, D‐GSPCAA and GSPCAA vs light illumination time, d) the evaporation rate and e) the evaporation efficiency of PPA, GPA, SPA, D‐GSPCAA and GSPCAA under the light irradiation, f) the evaporation rate of pure water and GSPCAA vs light irradiation time, g) the effect of initial concentration on adsorption capacity for Pb^2+^ under the dark and light conditions, h) Langmuir and Freundlich isotherm models for the adsorption of Pb^2+^ under the dark and light conditions, i) Temkin isotherm model for the adsorption of Pb^2+^ under the dark and light conditions, j) the effect of experimental temperatures on adsorption capacity for Pb^2+^ under the dark condition, k) Van't Hoff plots (lnKe vs 1/T) for the adsorption of Pb^2+^ under the dark condition, l) the effect of contact time on adsorption capacity for Pb^2+^ under the dark and light conditions, m) pseudo‐first‐order and pseudo‐second‐order models for the adsorption of Pb^2+^ under the dark and light conditions, n) the effect of initial pH value on adsorption capacity for Pb^2+^ under the light condition, o) the effect of coexisting ions on adsorption capacity for Pb^2+^ under the light condition, and p) the effect of reusability on adsorption capacity for Pb^2+^ under the light condition.

Subsequently, to more directly illustrate the enhancement of the photothermal properties on Pb^2+^ adsorption properties of all aerogels, batch adsorption tests were conducted in 50 mL of Pb^2+^ solution (200 mg L^−1^) under the dark and light conditions. The water temperature under the dark condition was controlled at 32.0 °C, which was the same as that under the light irradiation. For comparison, the adsorption under the light condition was also used as the above operation, except for the light irradiation. As shown in **Figure** **4**a, 5 mg (2 × 2 cm^2^) of GSPCAA was self‐floated on Pb^2+^ solution and irradiated simultaneously with 1 kW m^−2^ simulated light irradiation. Obviously, Pb^2+^ adsorption capacities of GSPCAA, D‐GSPCAA, SPA, and GPA under the light irradiation were all greater than those under the dark condition, confirming that light irradiation could enhance the adsorption capacities of Pb^2+^ on lignin‐based aerogels due to the addition of GO and SL (Figure 4b). This enhancement originated from irradiation‐induced thermal localization on lignin aerogels, concurrently accelerating Pb^2+^ diffusion/mass transfer kinetics and optimizing adsorption thermodynamics.^[^ ^52^ ^]^ It was observed that the adsorption capacity and removal efficiency of GSPCAA under the light condition were the best of 244.8 mg g^−1^ and 73.5%, respectively. On one hand, the strategic incorporation of oxygen/sulfur‐functional groups within GSPCAA's framework generated abundant binding sites.^[^ ^62^, ^63^ ^]^ On the other hand, the directional freezing process efficiently increased the specific surface area of GSPCAA, enhancing Pb^2+^ accessibility and thereby boosting adsorption efficacy.^[^ ^8^, ^64^ ^]^ Particularly, as compared with D‐GSPCAA, the ordered and dense structure of GSPCAA promoted diffusion and mass transport in solution as evidenced by the results of the solar water evaporation experiments, which also might be more favorable for the adsorption of Pb^2+^. As shown in Figure 4c, the water mass change of GSPCAA was much faster than that of pure water and other aerogels. After calculation, GSPCAA displayed the 3.24 kg m^−2^ h^−1^ of evaporation rate and remained stable over a long time (Figures 4d,e). In addition, the evaporation efficiency of GSPCAA was as high as 93.16%, achieving significant evaporation rates and high energy efficiency relative to recent materials (Figure 4f). All in all, GSPCAA presented the vertically oriented multiscale structure, excellent photothermal property, and outstanding Pb^2+^ adsorption capacity, which could be further used for the subsequent adsorption experiments.

### 2.5 Adsorption Performance of GSPCAA

To further obtain the Q_max_ of GSPCAA for Pb^2+^, adsorption experiments were performed at varying initial concentrations under dark and light conditions, respectively. As shown in Figure 4g, Pb^2+^ adsorption capacity by GSPCAA gradually rose with increasing initial concentration. When Pb^2+^ concentration reached 200 mg L^−1^, the adsorption equilibrium gradually began to occur. This was attributable to a greater concentration gradient on both sides of the GSPCAA surface within the low concentration ranged of 5–100 mg L^−1^, which facilitated the rapid adsorption of Pb^2+^ onto the active functional groups of GSPCAA.^[^ ^1^, ^8^ ^]^ However, the dosage of GSPCAA was fixed, leading to a limited number of its active functional groups. With further increase in initial concentration, the adsorption equilibrium was rapidly reached. At this point, most of the active functional groups had been effectively utilized, resulting in the adsorption amount of GSPCAA remaining basically unchanged.^[^ ^65^, ^66^ ^]^ Adsorption isotherm models are commonly used to analyze the interaction mechanism. As shown in Figures 4h,i and Table S1 (Supporting Information), the adsorption data of GSPCAA for Pb^2^⁺ were fitted using three nonlinear adsorption isotherm models. Obviously, the Langmuir model was demonstrated to be more suitable for describing the Pb^2^⁺ adsorption process by GSPCAA based on R^2^ value, indicating monolayer adsorption on the surface of GSPCAA.^[^ ^8^, ^67^ ^]^ According to calculations, the Q_max_ of GSPCAA for Pb^2^⁺ under the light irradiation reached 315.8 mg g^−1^, which was 1.3 times higher than that under the dark conditions (246.9 mg g^−1^), and significantly surpassed most previously reported biomass‐based adsorbents (Table S2, Supporting Information). This result further confirmed that solar irradiation could substantially enhance the Pb^2^⁺ adsorption capacity of GSPCAA. Comprehensively, it could be attributed to three main factors: 1) the efficient lignin sulfonation modification provided GSPCAA with abundant adsorption active sites, 2) the oriented layered structure and excellent hydrophilicity of GSPCAA synergistically constructed abundant oriented and hydrophilic channels, enabling Pb^2^⁺ to easily diffuse into its interior and achieving maximum utilization of adsorption active sites, 3) GSPCAA displayed superior photothermal property that enabled localized thermal hotspot formation under the light irradiation, further enhancing its Pb^2^⁺ adsorption efficiency.^[^ ^1^, ^68^ ^]^

Figure 4j presents the adsorption performance relationship between GSPCAA and Pb^2^⁺ under the different contact times at dark and light conditions. Whether under the dark or light conditions, Pb^2^⁺ adsorption process by GSPCAA gradually transitioned from the rapid adsorption stage to an equilibrium adsorption stage with increasing contact time. It was attributed to the abundance of active functional groups on GSPCAA's surface during the initial experimental phase, which provided sufficient adsorption sites and enabled rapid adsorption capacity enhancement.^[^ ^69^, ^70^ ^]^ However, as contact time prolonged, the active sites on GSPCAA became progressively occupied by Pb^2^⁺, ultimately leading to the emergence of the saturation adsorption phenomenon. Based on the above experimental data, nonlinear fitting curves obtained from pseudo‐first‐order and pseudo‐second‐order kinetic models are shown in Figure 4k, and the detailed fitting parameters are presented in Table S3 (Supporting Information). Comparative analysis revealed R^2^ values of 0.98 (dark, pseudo‐first‐order), 0.98 (light, pseudo‐first‐order), 0.99 (dark, pseudo‐second‐order), and 0.99 (light, pseudo‐second‐order) for GSPCAA's adsorption kinetics under the dark and light conditions. Pseudo‐second‐order kinetic model demonstrated superior fitness for describing Pb^2^⁺ adsorption on GSPCAA, indicating that chemical adsorption occurred on the surface of GSPCAA. Furthermore, K value under the light irradiation (17.36 × 10^−2^ g mg^−1^h^−1^) was significantly higher than that in the dark condition (13.94 × 10^−2^ g mg^−1^h^−1^), suggesting that solar irradiation effectively enhanced the adsorption capacity of GSPCAA. It indicated that GSPCAA could generate the local microthermal environment under the light condition, thereby accelerating the Pb^2^⁺ diffusion and mass transfer within the GSPCAA matrix.^[^ ^69^, ^70^ ^]^

To better elucidate the intrinsic mechanism underlying the significantly enhanced Pb^2^⁺ adsorption performances of GSPCAA under the light irradiation, we further investigated the relevant adsorption thermodynamic models. Figure 4l displayed the effect of temperatures on GSPCAA's Pb^2^⁺ adsorption capacity under the dark conditions. As the temperature increased at identical initial Pb^2^⁺ concentrations, both the equilibrium adsorption capacity and adsorption rate constant (K) of GSPCAA exhibited progressive enhancement (Table S4, Supporting Information). Especially, the superior adsorption performances of Pb^2^⁺ at elevated temperatures also suggested excellent thermal stability of GSPCAA.^[^ ^71^, ^72^ ^]^ Moreover, all adsorption processes at different adsorption temperatures could be effectively modeled using the Langmuir adsorption isotherm, confirming monolayer adsorption characteristics. Therefore, calculations revealed that GSPCAA achieved the Q_max_ of 315.8 mg g^−1^ at 328 K. Then, thermodynamic parameters including Gibbs free energy (ΔG, kJ mol^−1^), enthalpy change (ΔH, J mol^−1^), and entropy change (ΔS, J (mol·K)^−1^) were calculated for all adsorption processes using thermodynamic models. As shown in Figure 4m and Table S5 (Supporting Information), ΔG < 0 indicated that all adsorption processes were spontaneous. ΔG decreased with increasing adsorption temperature, suggesting that higher adsorption temperatures enhanced GSPCAA's adsorption capacity.^[^ ^73^, ^74^ ^]^ Conversely, the positive ΔH value (> 0) confirmed the irreversible endothermic nature of the adsorption process, while the positive ΔS value (> 0) indicated the increased system entropy during the adsorption process.^[^ ^73^, ^74^ ^]^ In summary, these results demonstrated that Pb^2^⁺ adsorption on GSPCAA followed an endothermic nature, further corroborating that the localized thermal microenvironment induced by illumination was also the primary factor enhancing Pb^2^⁺ adsorption capacity.

Figure 4n illustrates the effect of different initial pH values of GSPCAA under the light irradiation. It was found that the adsorption capacity initially increased sharply with the pH elevation but gradually declined beyond optimal ranges. The maximum Pb^2^⁺ adsorption capacity of 244.8 mg g^−1^ was achieved at pH 5.0, demonstrating GSPCAA's significant application potential for Pb^2^⁺ removal from weakly acidic wastewater. At low pH (<5.0), excessive H⁺ occupied adsorption sites while protonation of ─SO_3_^−^, ─COOH and ─OH groups induced positive surface charges, repelling cationic Pb^2^.^+[^ ^75^, ^76^ ^]^ Conversely, Pb^2^⁺ precipitation as Pb(OH)_2_ occurred above pH>5.0, where large ion radii and limited coordination sites restrict adsorption efficiency.^[^ ^75^, ^76^ ^]^ As a result, pH 5.0 was identified as the optimal adsorption condition via comprehensive parameter optimization.

Industrial wastewater from real‐world applications typically contains various interfering ions. Hence, the adsorption selectivity of GSPCAA is a key parameter for evaluating its applicability. We investigated the effect of coexisting metal ions on the adsorption selectivity of Pb^2^⁺ in a binary solute system (Figure 4o), such as Pb^2^⁺/Co^2^⁺, Pb^2^⁺/Mg^2^⁺, Pb^2^⁺/Cd^2^⁺, Pb^2^⁺/Cu^2^⁺, and Pb^2^⁺/Zn^2^⁺. Notably, GSPCAA exhibited superior Pb^2^⁺ adsorption performances under the light irradiation, significantly outperforming its adsorption capacities for co‐existing ions, thereby demonstrating enhanced Pb^2^⁺ selectivity. Besides, to quantitatively analyze this excellent adsorption selectivity, the distribution coefficient (K_d_) was also compared for Pb^2^⁺, Co^2^⁺, Mg^2^⁺, Cd^2^⁺, Cu^2^⁺, and Zn^2^⁺ binding affinity under the light irradiation. As shown in Table S6 (Supporting Information), K_d_ values of Pb^2^⁺, Co^2^⁺, Mg^2^⁺, Cd^2^⁺, Cu^2^⁺ and Zn^2^⁺ were 3.91 × 10⁵, 2.45 × 10^3^, 2.32 × 10^3^, 3.87 × 10^3^, 9.23 × 10^2^ and 6.87 × 10^2^ mL g^−1^, respectively. The significantly higher binding affinity of GSPCAA for Pb^2^⁺ compared to other metal ions confirmed its exceptional Pb^2^⁺ selectivity, primarily attributed to its abundant ─SO_3_^−^, ─COOH and ─OH groups. Meanwhile, this adsorption selectivity phenomenon could also be explained by Pearson's hard/soft acid/base (HSAB) theory. Based on this theory, metal ions acting as hard (soft) acids tend to form relatively stable complexes with hard (soft) ligands. From the acid/base classification, Pb^2^⁺ has been regarded as a softer acid than Co^2^⁺, Mg^2^⁺, Cd^2^⁺, Cu^2^⁺, and Zn^2^⁺.^[^ ^77^ ^]^ Thus, based on this assumption, the S/O‐containing ligands as soft bases in GSPCAA possessed a better affinity for the soft metal Pb^2+^ over Co^2^⁺, Mg^2^⁺, Cd^2^⁺, Cu^2^⁺, and Zn^2^⁺.^[^ ^8^ ^]^ Consequently, GSPCAA with outstanding selectivity to Pb^2^⁺ was suitable for the practical wastewater treatment process.

As shown in Figure 4p, ten adsorption‐desorption regeneration cycles were conducted on GSPCAA. Under the light irradiation, the adsorption performance of GSPCAA for Pb^2^⁺ did not significantly decrease with increasing cycle numbers. After 10th cycle, the adsorption performances of GSPCAA remained above 86% of the initial values, demonstrating that GSPCAA had good reusability and excellent stability. The slight decrease might be due to the loss of some active sites on the adsorbent surface and the low desorption efficiency during the regeneration process.^[^ ^67^, ^78^ ^]^

### 2.6 The Possible Adsorption Mechanism of GSPCAA

**Figure 5:**
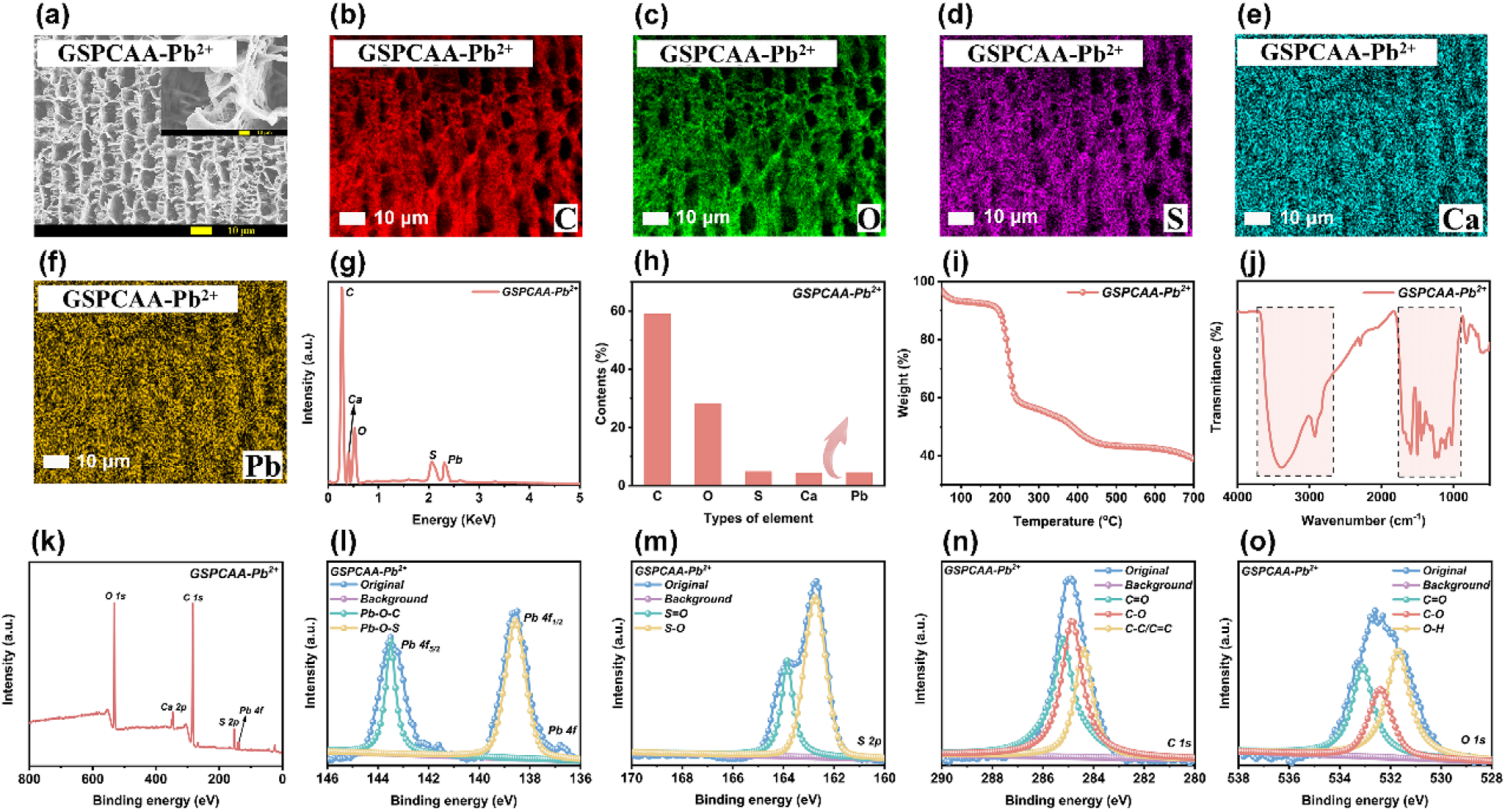
Characterization of GSPCAA‐Pb^2+^. a) SEM image of GSPCAA‐Pb^2+^, b–f) EDS elemental mappings of GSPCAA‐Pb^2+^, g) EDS pattern of GSPCAA‐Pb^2+^, (h) EDS results of GSPCAA‐Pb^2+^, i) TGA curve of GSPCAA‐Pb^2+^, j) FT‐IR spectra of GSPCAA‐Pb^2+^, k) XPS survey spectra of GSPCAA‐Pb^2+^, l) Pb 4f high‐resolution spectra of GSPCAA‐Pb^2+^, m) S 2p high‐resolution spectra of GSPCAA‐Pb^2+^, n) C 1s high‐resolution spectra of GSPCAA‐Pb^2+^, and o) O 1s high‐resolution spectra of GSPCAA‐Pb^2+^.

To systematically investigate the mutual adsorption mechanism of GSPCAA for Pb^2^⁺, we employed SEM, EDS, TGA, FTIR and XPS techniques to conduct an in‐depth analysis of the GSPCAA‐Pb^2^⁺. As shown in **Figure** **5**a, GSPCAA‐Pb^2^⁺ retained its vertically aligned 3D layered structure, indicating its excellent structural stability in an acidic environment (pH 5.0). Meanwhile, EDS analysis results (Figures 5b–h) revealed that Pb^2^⁺ was uniformly distributed across the vertical cross‐section of GSPCAA with a consistent content, providing direct evidence for the efficient adsorption of Pb^2^⁺ by GSPCAA. This uniform distribution suggested that Pb^2^⁺ could effectively diffuse into the internal structure of GSPCAA through the interlayer gaps, resulting in its excellent adsorption performances. In addition, TGA curves results (Figure 5i) showed that the final residual mass of GSPCAA‐Pb^2^⁺ reached 39.72%, significantly higher than the 29.31% residual mass before Pb^2+^ adsorption.^[^ ^66^, ^79^, ^80^ ^]^ This change also confirmed the successful adsorption of Pb^2^⁺. Figure 5j displays the FT‐IR spectra of GSPCAA‐Pb^2^⁺. Obviously, the stretching vibration characteristic peak of ─OH in GSPCAA‐Pb^2^⁺ shifted significantly from 3420 to 3413 cm^−2^, demonstrating that the ─OH functional groups of GSPCAA interacted with Pb^2^⁺ by electrostatic attraction during the adsorption process.^[^ ^66^, ^79^ ^]^ Additionally, the intensity of the ‐COOH peak decreased, and it shifted from 1717 to 1706 cm^−2^, indicating that the ─COOH functional groups also participated in the adsorption process.^[^ ^81^, ^82^ ^]^ At the same time, the significant shifts in the S═O (from 1261 to 1245 cm^−2^) and S─O (from 915 and 887 cm^−2^) characteristic peaks suggested that the ─SO_3_^−^ groups in GSPCAA exhibited certain coordination interactions with Pb^2^⁺.^[^ ^67^, ^78^ ^]^ XPS analysis further elucidated the adsorption mechanism. As shown in Figure 5k, the full‐spectrum XPS of GSPCAA‐Pb^2^⁺ exhibited the distinct Pb4f characteristic peak, confirming the successful loading of Pb^2^⁺ onto the surface of GSPCAA. The high‐resolution Pb4f spectrum revealed that the Pb 4f_1/2_ and Pb 4f_5/2_ characteristic peaks appeared at 138.65 and 143.55 eV, respectively, indicating that GSPCAA primarily adsorbed Pb^2^⁺ through chemical adsorption, with Pb existing mainly in ionic form on the material's surface (Figure 5l). The high‐resolution S 2p spectra showed that, compared to untreated GSPCAA, S═O and S─O characteristic peaks of GSPCAA‐Pb^2^⁺ shifted to 163.78 and 162.57 eV, respectively, further proving that ‐SO_3_^−^ was the primary active adsorption site (Figure 5m).^[^ ^47^, ^68^ ^]^ Moreover, the high‐resolution C 1s and O 1s spectra of GSPCAA‐Pb^2^⁺ displayed that the characteristic peaks of C═O, C─O and C─C/C═C shifted to 285.43, 284.81, and 283.21 eV, respectively, while the C═O, C─O, and O─H peaks shifted to 533.03, 532.38, and 531.88 eV, respectively (Figures 5n,o).^[^ ^1^, ^83^, ^84^ ^]^ These peaks shifts indicated that the oxygen‐containing functional groups in GSPCAA displayed significant interactions with Pb^2^⁺. These analyses collectively demonstrated that Pb^2^⁺ adsorption was predominantly governed by dual mechanisms: electrostatic attraction and coordination interactions.

### 2.7 Characterization and Triboelectric Property of GSPCAA‐Pb^2+^

**Figure 6:**
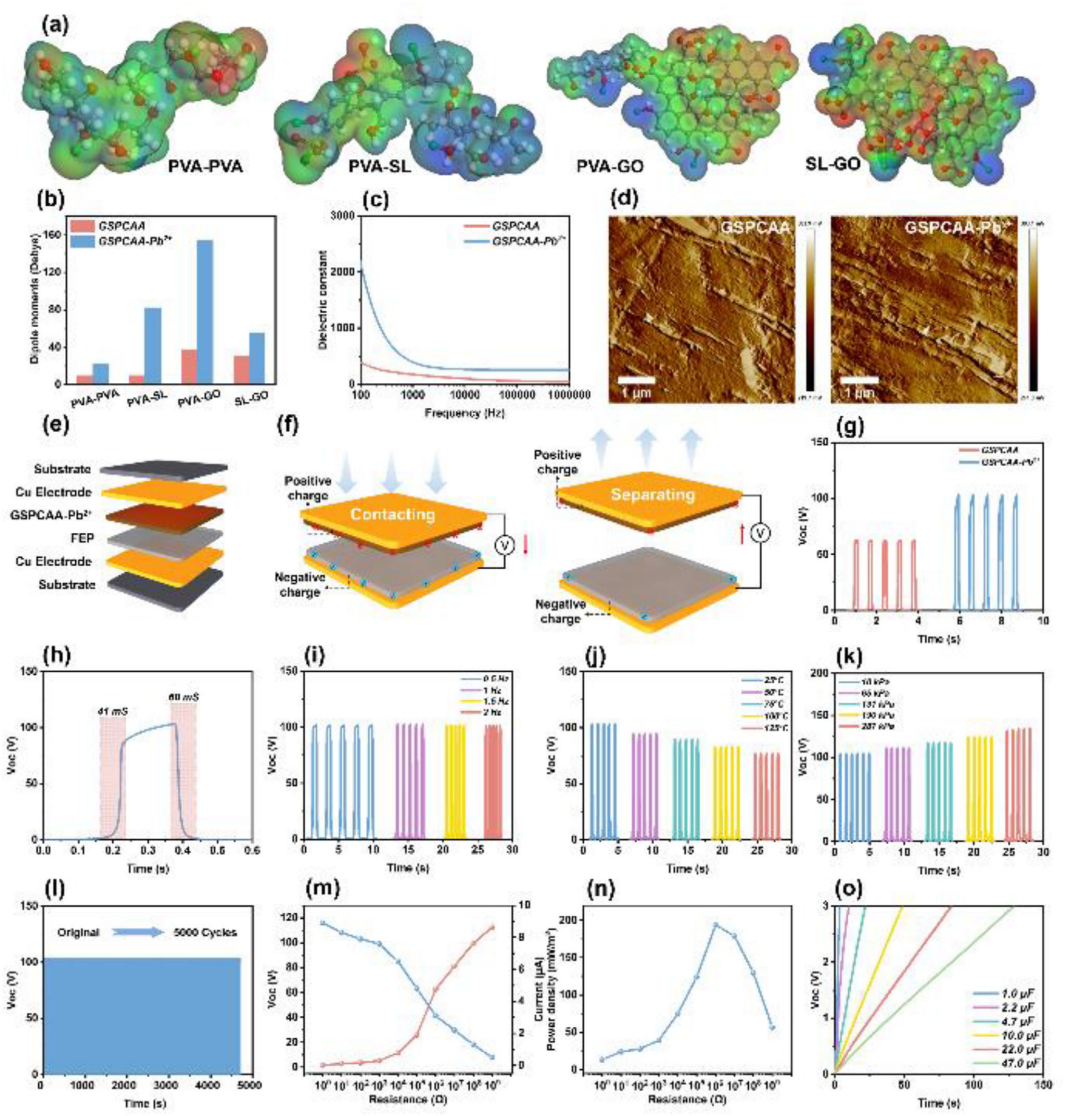
Characterization and triboelectric properties of GSPCAA‐Pb^2+^. a) The electrostatic potential distribution of the four pairs of components in the GSPCAA‐Pb^2+^ based on the DFT optimized structures, b) the dipole moments of the four pairs of components in the GSPCAA and GSPCAA‐Pb^2+^ based on the DFT optimized structures, c) dielectric constants of GSPCAA and GSPCAA‐Pb^2+^, d) AFM phase images of GSPCAA and GSPCAA‐Pb^2+^ at the same magnification, e) construction of the triboelectric nanogenerator, f) operating principles of the triboelectric nanogenerator, g) comparison of Voc of GSPCAA and GSPCAA‐Pb^2+^, h) GSPCAA‐Pb^2^ response and recovery time, i) comparison of Voc of GSPCAA‐Pb^2+^ in different frequency, j) comparison of Voc of GSPCAA‐Pb^2+^ in different temperature, k) comparison of Voc of GSPCAA‐Pb^2+^ in different pressure, l) the stability and durability test of GSPCAA‐Pb^2+^, m) Voc and Isc of GSPCAA‐Pb^2+^ with various external resistances, n) power density of GSPCAA‐Pb^2+^ with various external resistances, and o) charging curves of GSPCAA‐Pb^2+^ for different capacitance values.

In order to further achieve the maximal resource utilization of discarded adsorbents and better support the global transition to a low‐carbon society, after ten cycles of regeneration, GSPCAA‐Pb^2+^ was processed and applied for lignin‐based TENG, ultimately realizing the high‐value utilization of lignin‐based adsorbent materials in a complete closed loop. As shown in **Figure** **6**a, as compared with the original structural state (Figure S8, Supporting Information), the electron cloud volume in the red circle significantly increased after Pb^2^⁺ adsorption. In this way, due to the hybridization of the frontier *π* orbitals of the conjugated ligand with the d orbitals of the Pb metal, the delocalization of electrons on the structural framework was triggered, resulting in expanded electron clouds and enhanced molecular polarization, thereby promoting charge transfer between the electrodes during electrification.^[^ ^41^, ^85^ ^]^ Typically, the polarity of the molecule can also be directly expressed through its dipole moment, which is simulated by Gaussian software. However, due to the highly aggregated and complex nature of GSPCAA‐Pb^2^⁺, it is difficult to directly calculate the overall dipole moment of GSPCAA‐Pb^2^⁺. Therefore, four main connection modes of GSPCAA‐Pb^2^⁺ were selected as fixed models. Clearly, after Pb^2^⁺ adsorption, the molecular dipole moments of all four models were significantly enhanced (Figure 6b). This phenomenon could be explained as the mutual hybridization between the Pb metal and the molecular structure, which increased the distance between the positive and negative charge centers, thereby leading to the separation of electrons from the basic framework and enhancing the molecular dipole moment.^[^ ^61^, ^86^ ^]^ Additionally, the dielectric constant is directly proportional to the molecular dipole moment.^[^ ^87^, ^88^ ^]^ The dielectric properties can influence the electron affinity, thereby determining the charge density generated on the frictional electrodes. As shown in Figure 6c, the frequency‐dependent dielectric properties of GSPCAA and GSPCAA‐Pb^2+^ were measured within the frequency range of 1 kHz to 1 MHz. It could be observed that after Pb^2+^ adsorption onto GSPCAA, its dielectric properties exhibited a significant improvement trend. Subsequently, to better quantify the surface potential of the material after adsorption, Figure 6d shows the phase images of GSPCAA and GSPCAA‐Pb^2+^ obtained by Atomic Force Microscopy (AFM) at the same magnification. As compared with the original GSPCAA, GSPCAA‐Pb^2^⁺ exhibited a higher surface potential, indicating the significant expansion of molecular polarity, which might enhance the voltage output performance (Voc) of the materials.

The assembly of GSPCAA‐Pb^2^⁺ in TENG is shown in Figure 6e, where GSPCAA‐Pb^2^⁺ served as the positive triboelectric layer, and fluorinated ethylene propylene (FEP) acted as the negative triboelectric layer. In the whole working cycle, the negatively charged FEP first comes into contact with GSPCAA‐Pb^2^⁺, causing surface electron transfer.^[^ ^53^, ^54^ ^]^ After that, gradual compression leads to structural deformation, during which more electrons are generated and extracted within the structure, ultimately producing a stable triboelectric output signal (Figure 6f). As shown in Figure 6g and Figure S9 (Supporting Information), as compared with GSPCAA (59.6 V, 1.2 µA, and 22.3 nC), the Voc of the TENG fabricated with GSPCAA‐Pb^2^⁺ could be significantly increased to 101.3 V, 2.7 µA, and 49.5 nC. Meanwhile, Figure 6h shows the specific response time and recovery time of GSPCAA‐Pb^2^⁺ as a TENG, which were only 41 and 68 mS, respectively, both of which were below the human perceivable delay time (100 mS), making it an ideal choice for practical applications.^[^ ^89^, ^90^ ^]^ Furthermore, the Voc of the TENG fabricated with GSPCAA‐Pb^2^⁺ remained stable and unchanged under frequencies below 2.0 Hz, which also indicated that this material was sufficient to meet the demands of most daily human activities (Figure 6i). As shown in Figure 6j, due to the certain thermal stability of GSPCAA‐Pb^2^⁺, it could maintain excellent energy harvesting efficiency at various temperatures. When the temperature increased to 125 °C, the Voc still reached 75.8 V. Notably, based on the excellent mechanical performances of the GSPCAA‐Pb^2^⁺, the Voc of the fabricated TENG was positively correlated with the applied pressure. When the pressure improved from 10 to 287 kPa, the Voc could continuously increase from 101.3 to 129.6 V (Figure 6k). Durability is one of the key factors in evaluating the practical applications of the TENG. As shown in Figure 6l, after 5000 cycles, the GSPCAA‐Pb^2^⁺’s TENG could still maintain stable and continuous Voc performances. As shown in Figure 6m, under the same test conditions, the Voc and IµA of the TENG fabricated with GSPCAA‐Pb^2^⁺ were recorded using different load resistors. Clearly, the Voc increased with the increase of the external load resistance, while the IµA exhibited the opposite trend due to Ohmic losses. The output power density was calculated according to P = U^2^/R, and the maximum instantaneous power density of GSPCAA‐Pb^2+^ could reach 197.45 mW m^−^^2^ (Figure 6n). At last, the charging capacity tests were also conducted. As shown in Figure 6o, the Voc reached 3 V in the fastest 3 s (with a capacity of 1.0 µF), and it could also be charged with capacitors of various capacities (from 1.0 to 47.0 µF).

### 2.8 Application in Smart Home Control

**Figure 7:**
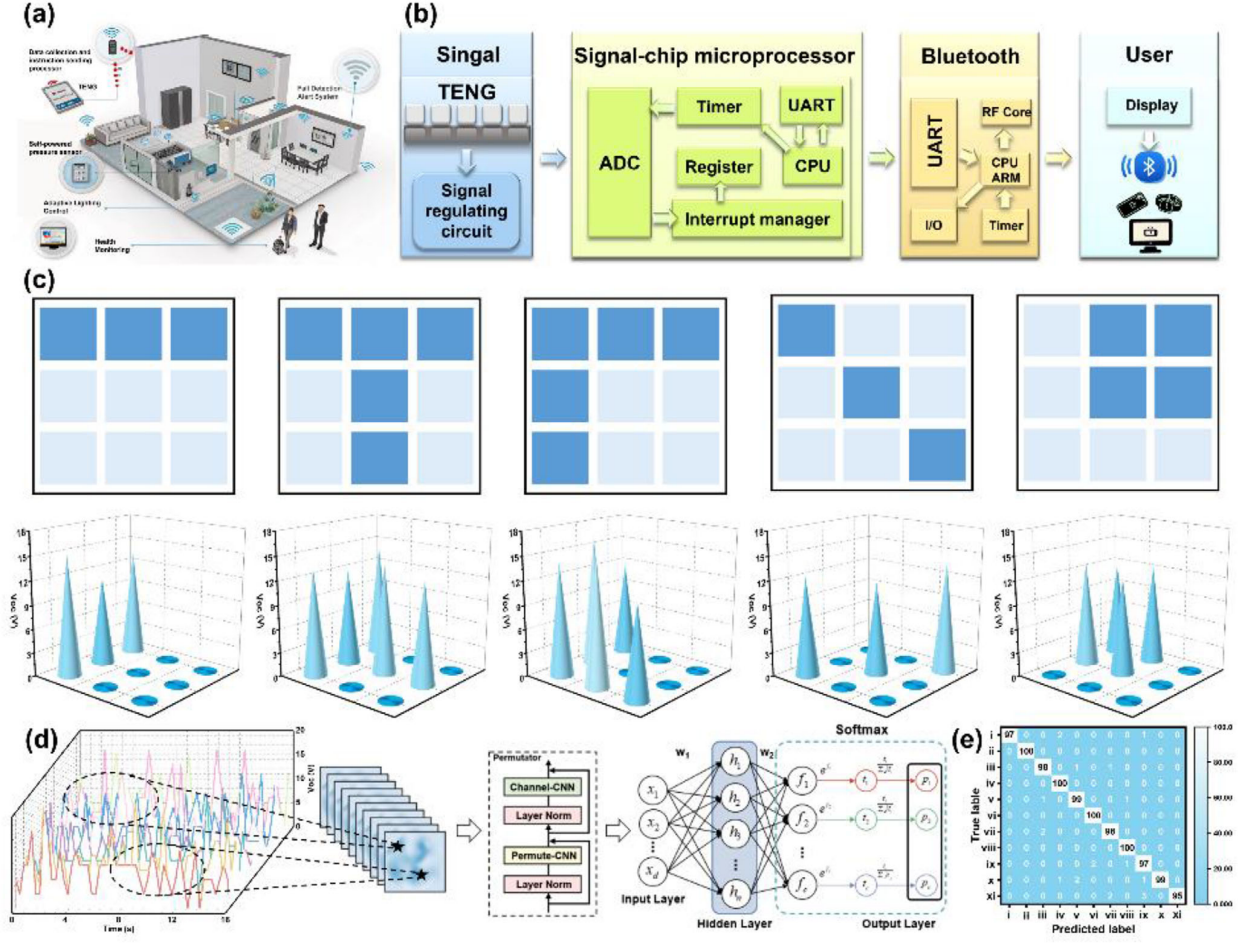
Practical applications of the GSPCAA‐Pb^2+^ triboelectric nanogenerator. a) Schematic diagram of triboelectric nanogenerator in smart home control applications, b) schematic diagram of wireless signal transmission process, c) recognition of different graphics by sensor arrays, d) schematic diagram of the overall workflow of the identification module, and e) the confusion matrix for identification accuracy of eleven samples.

In the era of “Internet of Things,” the future development trend is the acceleration and intelligence of the signal transmission. Thus, in the field of smart homes, TENG technology can be fully utilized to achieve real‐time data acquisition, transmission, and intelligent analysis without an external power source (**Figure** **7**a). In this work, leveraging the excellent mechanical performances and outstanding TENG output performances of GSPCAA‐Pb^2^⁺, a 3 × 3 array of GSPCAA‐Pb^2+^ was fabricated as an intelligent self‐powered sensor. As shown in Figure 7b, when the array was subjected to impact, the self‐powered sensor generated a real‐time signal output. Subsequently, the real‐time signal was transmitted to the signal adjustment circuit composed of transistors and resistors to adjust the signal amplitude. Next, the signal was further transmitted to the analog‐to‐digital converter (ADC) of the microcontroller to convert the multi‐channel analog signals into digital signals. Finally, the digital signal was transmitted to the central processing unit for processing and wirelessly transmitted to a smartphone terminal via the Bluetooth module for real‐time detection. Figure 7c illustrates the distinct Voc signals obtained by the 3 × 3 matrix through effectively distinguishing the loading and unloading regions of various objects, as well as the electric signal heatmap generated after normalization. Notably, these signal differences could intuitively reflect the size of the loaded object and its pressure distribution characteristics, thereby aiding in the precise identification of human location and motion trajectory in a smart home environment, thereby ultimately providing clear perceptual information for the occupants. Considering that different loaded objects possess unique electric signal characteristics, we also proposed a real‐time recognition model based on the convolutional neural network (CNN) for intelligent behavior classification. Figure 7d provides a detailed flowchart of the learning model, and the specific parameters are listed in Table S8 (Supporting Information). The network model can be trained from scratch using a series of the Voc signals directly as input data or by converting them into input images. Through effectively extracting deep local features from low‐level to high‐level convolutional kernels, the model ultimately achieves good spatial invariance. Figure 7e displays the confusion matrix of classification results for 11 different objects, where each row represents test samples of actual categories, and each column corresponds to predicted categories. It could be found that the confusion matrix achieved an overall recognition accuracy of 98.5%. Each behavior exhibited varying recognition accuracy along the diagonal. Therefore, this result demonstrated that, in complex scenarios, the 3 × 3 self‐powered sensor array could perfectly integrate with the deep learning classification models, enabling precise real‐time monitoring and object differentiation with the excellent practical potential.

## 3 Conclusion

In this study, we established a two‐step strategy to achieve the simple synthesis of lignin‐based aerogel with excellent adsorption performances, and then reused it as lignin‐based TENG. Firstly, sulfonation was performed on the lignin structure to increase the number of active adsorption sites and improve the water solubility of the polymer. Subsequently, high‐strength GSPCAA with vertically aligned channels was prepared using directed freezing and solution immersion methods. Batch experiments demonstrated that the synthesized GSPCAA exhibited a highly efficient adsorption capacity of Pb^2^⁺ with the Q_max_ of 315.8 mg g^−1^. The single‐layer chemical adsorption process could be confirmed by the adsorption isotherm and kinetic models. Thermodynamic modeling confirmed the endothermic and spontaneous nature of all processes. More significantly, to further achieve high‐value applications of discarded adsorbents, a novel lignin‐based TENG was prepared using GSPCAA‐Pb^2^⁺ as the primary material. Based on this material, it displayed a fast response time (41 and 68 mS), excellent electric signal output performances (101.3 V, 2.7 µA, and 49.5 nC), outstanding environmental stabilities (various frequency, temperature, and pressure) and durable working cycle life (stable operation for 5000 cycles). In addition, a 3 × 3 self‐powered array was also designed to achieve real‐time visualization and accurate identification of pressure distribution for different loaded objects. Meanwhile, it was combined with deep learning model algorithms, ultimately achieving an overall recognition accuracy of 98.5% in the classification and identification of 11 different objects, thereby fully demonstrating its practical application potential in the smart home field. Although this proposed method still had certain limitations in technical and economic aspects, it significantly improved the research on the recycling and reuse of the discarded adsorbents. In summary, this work provides new insights into the multifunctional application of lignin resources.

## 4 Experimental Section

The detailed experimental procedures and characterization results are provided in the Supporting Information.

Statistical analysis was performed on the data, which was presented as the standard deviation (SD) ± mean value. Error bars in the data plots indicated the SD calculated from at least five tests conducted on each sample. Data processing was carried out using Origin 2021. If not noted otherwise, each experiment was repeated five times.

## Conflict of Interest

The authors declare no conflict of interest.

## Author Contributions

B.D. contributed to writing—review and editing, supervision, funding acquisition, and conceptualization. Y.Y. was responsible for writing the original draft, while L.H. carried out the formal analysis. Y. X. contributed to conceptualization and visualization. X.W. worked on methodology, validation, and investigation, and J.Z. contributed to data curation and supervision. X.J. was involved in methodology and conceptualization, whereas L. S. contributed to investigation and project administration.

## Supporting information



Supporting Information

## Data Availability

The data that support the findings of this study are available from the corresponding author upon reasonable request.
