# Supplementary material for: From Industrial Waste to Multistage Applications: Ultralight Lignin‐Based Aerogel with Situ Vertically Oriented Structure for Photothermal‐Assisted Pb2+ Adsorption in Wastewater and Reuse as Efficient Output and Stability Triboelectric Materials
Source: Adv Sci (Weinh). 2025 Sep 30;12(47):e13337. doi: 10.1002/advs.202513337 (PMC12713064; doi:10.1002/advs.202513337)
Supplement: Supplementary file 1 — Supporting Information [file ADVS-12-e13337-s001.docx]

Supporting Information

**From Industrial Waste to Multistage Applications: Ultralight Lignin-Based Aerogel with Situ Vertically Oriented Structure for Photothermal-Assisted Pb^2+^ Adsorption in Wastewater and Reuse as Efficient Output and Stability Triboelectric Materials**

Boyu Du ^1,2*^, Yuxin Yang ^2^, Lingjing Huang ^2^, Yuxuan Xie ^2^, Xing Wang ^3*^, Jinghui Zhou ^3^, Xingxiang Ji ^1^ and Lupeng Shao ^1*^

^1^ Key Laboratory of Pulp and Paper Science & Technology of Ministry of Education, Qilu University of Technology (Shandong Academy of Sciences), Jinan 250353, China

2 Guangxi Key Laboratory of Clean Pulp & Papermaking and Pollution Control, School of Light Industry and Food Engineering, Guangxi University, Nanning, 530004, PR China

3 Liaoning Key Lab of Lignocellulose Chemistry and Bio Materials, Liaoning Collaborative Innovation Center for Lignocellulosic Biorefinery, College of Light Industry and Chemical Engineering, Dalian Polytechnic University, Dalian 116034, China

*Corresponding author. Address:

^1^ Key Laboratory of Pulp and Paper Science & Technology of Ministry of Education, Qilu University of Technology (Shandong Academy of Sciences), Jinan 250353, China

^2^ Guangxi Key Laboratory of Clean Pulp & Papermaking and Pollution Control, School of Light Industry and Food Engineering, Guangxi University, Nanning, 530004, PR China

^3^ Liaoning Key Lab of Lignocellulose Chemistry and Bio Materials, Liaoning Collaborative Innovation Center for Lignocellulosic Biorefinery, College of Light Industry and Chemical Engineering, Dalian Polytechnic University, Dalian 116034, China

This Supporting information includes the following parts:

Experimental Section

Supporting **Scheme S1**

Supporting **Figure S1** to **Figure S10**

Supporting **Table S1** to **Table S8**

**1. Experimental section**

**1.1 Materials**

Original lignin (OL) used was the industrial by-product from Shandong Longlive Bio-technology Co., Ltd., China. Hydrogen peroxide solution (H_2_O_2_, 30%) was purchased from Xin Ke Electrochemical Reagent Factory, China. Formaldehyde solution (CH_2_O, 37%), graphite powder, sodium sulfite (Na_2_SO_3_, AR), polyvinyl alcohol (PVA, AR), sodium hydroxide (NaOH, 96%) and hydrochloric acid (HCl, 36%-38%) were obtained from Shanghai Macklin Biochemical Co., Ltd., China. Lead nitrate (Pb(NO_3_)_2_), cobaltous nitrate (Co(NO_3_)_2_, AR, 98%), manganese nitrate (Mn(NO_3_)_2_), cadmium nitrate (Cd(NO_3_)_2_), copper nitrate (Cu(NO_3_)_2_), zinc nitrate (Zn(NO_3_)_2_), potassium bromide (KBr, ACS, 99%), potassium permanganate (KMnO_4_) and sodium hydroxide (H_2_SO_4_, AR, 36-38%) were purchased from Shanghai Aladdin Chemical Reagent Co., Ltd., China. Fluorinated ethylene propylene (FEP) film was provided by Polyfluorene New Material Science and Technology Co. All commercially available chemical reagents were used directly without further purification.

**1.2 Preparation of GO**

Graphene (GO) was prepared by modified Hummers methods [1]. Concentrated H_2_SO_4_ (23 mL) and graphite powder (1.0 g) were mixed in an ice bath and stirred for 30 min. KMnO_4_ (3.0 g) was then added to the mixture and stirred for 2 h at 35^o^C. Subsequently, deionized water (46 mL) was added and the stirring was continued for 15 min at 95^o^C. After that, the suspension was diluted with 60 mL deionized water. When the temperature cooled to 65^o^C with no bubbles being observed, H_2_O_2_ (5 ml) was added. Then the sediment was produced and collected by centrifugation. After washing to neutral, the product was lyophilized and named as GO.

**1.3** **Sulfonation of SL**

The sulfonation of OL was performed under alkaline condition. Typically, 1.0 g OL was slowly dissolved into 10.0 mL NaOH solution (2 wt%). 1.2 g CH_2_O and 1.2 g Na_2_SO_3_ were then added into the above system with continuous stirring. This system was subsequently heated and maintained at 80°C for 4 h to complete the sulfonation. After this, the resulting product was cooled, and purified by dialysis tubing with a molecular weight cut-off of 1000 Da. The sulfonated lignin (SL) was finally obtained by freeze-drying.

**1.4 Preparation of GSPCAA**

Lignin-based aerogel (GO/SL/PVA/Ca, GSPCAA) were fabricated by the directional freeze-casting combined with a mild ionic crosslinking process. In brief, 1.0 g of PVA and 0.5 g of SL were dissolved into the mixed solution containing 50 mL of GO dispersion liquid (5 mg/mL) by continuous mechanical agitation. The obtained mixture was then floated on the liquid nitrogen to accomplish the directional freeze-casting. After freeze drying, the resultant GSPCAA precursor was immersed into 2 wt% CaCl_2_ solution to perform a mild ionic crosslinking process. Finally, GSPCAA was obtained by washing with deionized water and freeze-drying process.

To explore the effect of directional freeze-casting process on the physicochemical properties of GSPCAA, disordered-GSPCAA (D-GSPCAA) was also fabricated. Typically, 1.0 g of PVA and 0.5 g of SL were dissolved into the mixed solution containing 50 mL of GO dispersion liquid (5 mg/mL) by continuous mechanical agitation. After that, the obtained mixture was directly freeze-dried, and then immersed into 2wt % CaCl_2_ solution to perform a mild ionic crosslinking process. Finally, D-GSPCAA was obtained by washing with deionized water and freeze-drying process.

Particularly, according to the contents of GO and SL, different samples were named as PPA (without GO and SL), GPA (without SL) and SPA (without GO).

**1.5 Adsorption experiments**

Firstly, Pb^2+^ solution (200 mg/L) was prepared. Then, 30 mg of the adsorbent was mixed in 50 mL Pb^2+^ solution. After adsorption process, the residual concentration of Pb^2+^ was detected by inductively coupled plasma optical emission spectrometry (ICP-OES, Avio 500, PerkinElmer, USA). The adsorption capacity (Q_e_, mg/g) and removal efficiency (R, %) were computed from Eq. (1) and Eq. (2), respectively.

$Q_{e}=\frac{(C_{0} - C_{e})V}{m}$ (1)

$R=\frac{(C_{0} - C_{e})}{C_{0}}*100\%$ (2)

where C_0_ and C_e_ (mg/L) are Pb^2+^ concentrations at initial and equilibrium, respectively, m (mg) is the weight of adsorbent, and V (mL) is the volume of Pb^2+^ solution.

The adsorption isotherms were mainly used to evaluate the adsorption equilibrium properties and describe how Pb^2+^ interacts with the adsorbent. The Langmuir, Freundlich and Temkin isotherm models were utilized to fit the adsorption data. The three models were shown in the following Eq. (3), Eq. (4) and Eq. (5), respectively [2, 3]:

$Q_{e}=\frac{Q_{\max}K_{L}C_{e}}{{1+k}_{L}C_{e}}$  (3)

$Q_{e}=K_{F}{C_{e}}^{1/n}$ (4)

$Q_{e}=B_{T}\ln K_{T}+B_{T}\ln C_{e}$ (5)

where Q_e_ and Q_max_ present the equilibrium adsorption capacity and the maximum adsorption capacity of Pb^2+^ on the adsorbent, respectively. K_L_ and K_F_ (L/mg) present the constant of the Langmuir model and Freundlich model, respectively. C_e_ is the concentration of Pb^2+^ at adsorption. 1/n is the heterogeneity factor. B_T_ (J/mol) and K_T_ (L/mg) refer to the Temkin isotherm constants.

In order to better analyze the adsorption mechanism and the adsorption rate, two widely-used kinetic types including the pseudo-first-order model and the pseudo-second-order model were utilized to analyze the adsorption process according to the Eq. (6) and Eq. (7), respectively [4, 5]:

$Q_{t}=(1-exp(-k_{1}t))$ (6)

$Q_{t}=\frac{k_{2}{Q_{e}}^{2}t}{{1+k}_{2}Q_{e}t}$ (7)

where k_1_ and k_2_ present the rate constants of the pseudo-first-order and the pseudo-second-order model, respectively. Q_t_ is the adsorption capacity at time t.

The thermodynamic parameters for Pb^2+^ adsorption by adsorbent were calculated by the following Eq. (8), Eq. (9) and Eq. (10), respectively [6, 7]:

$K_{e}=\frac{1000K_{L}\left( molecular weight of Pb \right)\mathrm{Adsorbate}^{0}}{\gamma}$ (8)

$\ln\left( K_{e} \right)=-\left( \frac{\Delta H}{R} \right)\frac{1}{T}+\frac{\Delta S}{R}$ (9)

$\Delta G=\Delta H-T\Delta S$ (10)

where K_e_ is the thermodynamic equilibrium constant that is dimensionless; K_L_ (L/mg) refers to the Langmuir models’ coefficient; γ is the coefficient of activity (dimensionless); [Adsorbate]^0^ is the standard concentration of the adsorbate (1 mol/L); T (K) represents the temperature of Pb^2+^ solution. R (8.314 J/mol/K) is the gas constant. ΔG (KJ/mol) is the stand Gibbs free energy. ΔH (KJ/mol) is the standard enthalpy change. ΔS (KJ/mol/K) is the standard entropy change.

In 50 mL Pb^2+^ solutions, a series of adsorption experiments under different initial pH value (1.0-7.0) were investigated in the initial concentration of 200 mg/L, respectively.

As for the experiments of adsorption affinity, the testing solutions of Pb^2+^, Co^2+^, Mg^2+^, Cd^2+^, Cu^2+^ and Zn^2+^ were first prepared by diluting the stock solutions of the six metal ions (1000 mg/L) with ultrapure water. Then, 0.05 g of adsorbent was placed into a 250-mL beaker and then 50 mL of the testing solution with individual Pb^2+^, Co^2+^, Mg^2+^, Cd^2+^, Cu^2+^ and Zn^2+^ were added, respectively. The initial concentration of each metal ions was about 5 mg/L, and the value of the solution pH was adjusted to 5.0 by using HCl (0.1 M) and NaOH (0.1 M). All beakers were covered by parafilm and shaken at 150 rpm under room temperature in a shaker. At a certain time interval, the samples were periodically withdrawn with a 5-mL pipette (Research plus, Eppendorf, Germany) and then filtered through a 0.45-µm nylon membrane (Beyotime Biotechnology, Shanghai, China). The filtrates were used for analyzing the residual concentrations of six metal ions. The control experiments were conducted under the identical conditions. For control experiments, 50 mL of Pb^2+^, Co^2+^, Mg^2+^, Cd^2+^, Cu^2+^ and Zn^2+^ solution was individual placed into a 250-mL flask without adding adsorbent. The distribution coefficient (K_d_) was calculated in the following Eq. (11):

$K_{d}=\frac{V[(C_{0}-C_{f})/C_{f}]}{M}$ (11)

where C_0_ (mg/L) and C_f_ (mg/L) are the initial and equilibrium concentrations of M^n+^ after adsorption, respectively. V (mL) is the solution volume and M (g) is the adsorbent amount. V/m ratio of 1000 mL/g was used in this experiment.

To study the effects of co-existing ions (Co^2+^, Mg^2+^, Cd^2+^, Cu^2+^ and Zn^2+^) of adsorbent, a series of adsorption experiments were performed by an initial concentration of 200 mg/L. Two type of heavy metal ions weight concentration ratio was 1:1 (equal concentrations of 200 mg/L for 48 h at pH=5.0 and 298 K).

To test the reusability of GSPCAA, GSPCAA adsorbed with Pb^2+^ (GSPCAA-Pb^2+^) was immersed in 0.1 M hydrochloric acid (HCl) for 600 min, followed by repeated washing with HCl, ethanol, and deionized water, to completely desorb the adsorbed Pb^2+^ from GSPCAA until the pH reached 7.0. Subsequently, the desorbed GSPCAA was dried at 50°C until constant weight, and the regenerated GSPCAA was further used in the next cycle of Pb^2+^ adsorption under the same conditions. The adsorption-desorption cycles were carried out ten times.

If not noted otherwise, each experiment was repeated for five times. All data were analyzed statistically and the error bars depicted 95% confidence intervals.

**1.6 Preparation and working principle of TENG**

TENG adopts a vertical contact mode, with GSPCAA or GSPCAA-Pb^2+^ as the positive triboelectric material, FEP film as the negative triboelectric material, double-sided Cu film as the electrode, and acrylic board as the supporting layer. The specific manufacturing steps include processing the acrylic support plate and fixing the positive and negative triboelectric materials, adhering the GSPCAA/GSPCAA-Pb^2+^ and FEP film to both sides of the supporting layer, connecting double-sided Cu electrodes to the back of the materials, and extending wires for electrical signal transmission. When the two layers come into contact, due to the difference in electron affinity, electrons transfer from the GSPCAA/GSPCAA-Pb^2+^ to the FEP film, resulting in the contact electrification phenomenon. Upon separation, charges remain on their respective surfaces, creating a potential difference. This potential difference drives electrons to flow through the external circuit, thereby generating electrical signals.

**1.7 Preparation of TENG array**

The GSPCAA-Pb^2+^ sample (thickness 100 μm) was first cut into square-shaped monomer components using a laser cutting machine and pasted on double-sided Cu film. Then, the double-sided Cu film was placed on square PDMS (thickness 3 mm) with a diameter of 10 cm. Finally, copper wires were pasted on double-sided Cu film to connect the self-powered sensor monomers into an array component.

**1.8 Collection of TENG performances**

The TENG performance was tested using an electrometer (Keithley 6514, USA) and a data acquisition card (NI-USB6259, USA), including voltage, current, and charge. Specifically, the positive and negative electrodes of the TENG were connected to the metal probes at both ends of the electrometer. A linear motor (Linmot E1100) was used to control the operating frequency of the TENG at 1 Hz with a separation distance of 15 cm (unless otherwise specified). The test conditions were maintained at a humidity of ≈40% RH and a temperature of 25°C (unless otherwise specified). A pressure sensor (HY Chuangan HYPS017Z, China) was placed at the bottom of the TENG to feedback the applied stress intensity. The sensor's accuracy was one-thousandth, with a data acquisition frequency of 33 Hz.


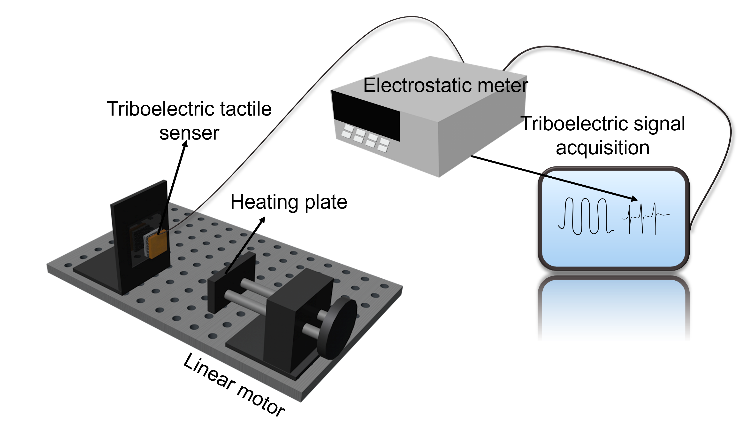


**Scheme S1** Schematic diagram of TENG performance testing.

**1.9 Characterizations**

**1.9.1 2D heteroniclear single quantum coherence (HSQC)**

2D-HSQC determinations for lignin were recorded on the Bruker AVAVCE III HD 400 MHz spectrometer at 25^o^C [8-11]. About 50 mg of fractionated lignin was dissolved in 0.5 mL of DMSO-*d6* (99.8% D). For quantitative 2D-HSQC spectra, the Bruker standard pulse program hsqcetgpsi2 was used for HSQC experiments. The spectral widths were 5000 Hz and 20000 Hz for the ^1^H and ^13^C-dimensions, respectively. The number of collected complex points was 1024 for ^1^H-dimension with a recycle delay of 1.5 s. The number of transients was 64 and 256 times increments were always recorded in the ^13^C-dimension. The ^1^JCH used was 145 Hz. Prior to Fourier transformation, the data matrixes were zero filled up to 1024 points in the ^13^C-dimension. Data processing was performed using standard Bruker Topspin-NMR software.

**1.9.2 Fourier transformation infrared spectroscopy (FT-IR)**

FT-IR analysis was performed on a Perkin-Elmer spectrophotometer. Briefly, 1.0 mg of lignin and 200 mg of KBr were ground and pressed to tablets. Each spectrum was scanned 32 times in the range of 4000-500 cm^-1^ with a resolution of 4 cm^-1^ in the transmission mode [12].

**1.9.3 Scanning electron microscope (SEM)**

A scanning electron microscope (SEM, JEOL JSM-7800F) was used to analyze the surface characteristics of GSPCAA. Before the SEM observation, the samples were sputtered with a thin layer of gold coating [13, 14].

**1.9.4 X-ray photoelectron spectroscopy (XPS)**

The chemical composition was studied by using XPS (ESCALAB 250Xi, Thermo Fisher). Initially the low resolution survey was performed to identify the elements present on the surface. Then, high resolution C 1s, O 1s, S 2p, Ca 2p and Pb 4f regions were analyzed to get the binding characteristics of lignin surface. Shirley background and Gaussiane Lorentzian functions were used for peak fitting [15].

**1.9.5 Zeta potentials**

The zeta potentials of samples were tested by a Particle Charge Detector (PCD-04 Travel). The samples were prepared with a concentration of 2.0 g/L, each sample was measured three times for average [16].

**1.9.6 Brunauer-Emmett-Teller (BET)**

BET specific surface area of samples were calculated from a multipoint BET analysis of nitrogen (N_2_) adsorption isotherms and N_2_ adsorption-desorption isotherm was performed using a Kubo-X1000 instrument (Beijing Builder Co. Ltd, China) by liquid N_2_ (-196^o^C). Before measurement, the sample was degassed by vacuum at 200^o^C for 5 h [17].

**1.9.7 Element analysis (EA)**

The contents of C, H, O, S and Ca of samples were determined by a Flash 2000 series CHNSO analyzer (Thermo Scientific, MA, USA) [18].

**1.9.8** **X-ray diffraction (XRD)**

The crystalline structure of the triboelectric film was measured on XRD (BRUKER D8 Discover A24A10, Germany) at a scanning rate of 5 ^o^/min [19].

**1.9.9 Thermal analysis (TGA)**

The thermal stability of samples and mass loss of samples were determined by Q500 thermogravimetric analyzer (TA Instruments). Approximately 10 mg sample in a small crucible were performed under a nitrogen atmosphere with temperature ranging from 25^o^C to 800^o^C at the heating rate of 10 ^o^C/min [20].

**1.9.10 Atomic force microscope (AFM)**

Adopted the dimension icon AFM of Brook company in USA. The Peak Force Quantitative Nano-Mechanics (PF-QNM) mode of the improved tap mode was used to take the Peak Force as the feedback signal, and the Peak Force between the probe and the sample was kept constant by the movement of the scanning tube, so as to obtain the morphology and mechanical properties of the sample [21].

**1.9.11 Mechanical test**

Tensile and compression tests were performed using an Instron 5300 universal testing machine. The strain rates for tensile and compression tests were 2 mm/min and 5 mm/min, respectively. The strain rate for cyclic compression was 5 mm/min [22].

**1.9.12 Contact angle experiment**

The contact angle experiment was used to analyze the hydrophilic and hydrophobic properties of materials by a KSV Instrument [23].

**1.9.13 Ultraviolet-visible spectroscopy (****UV-vis-NIR)**

Optical absorbance spectra of adsorbents were recorded in UV-vis-NIR spectrophotometer (Perkin-Elmer LAMBDA 950) [23, 24].

**1.9.14 Infrared thermal images**

The infrared thermal images were captured by an infrared thermal camera (Infiray-T2S+) [23, 24].

**1.9.15 Dielectric constants**

The dielectric constants were determined by an Agilent LCR meter (4990A) at room temperature by changing the frequency from 1k Hz to 1M Hz [25, 26].

**1.9.16 Solar Desalination experiment**

The illumination was provided by a controllable solar simulator (CME-SL500) [23]. The illumination density was measured by a photo radiometer (TENMARS, TM-207). The heating state and surface temperature of GSPCAA were monitored by an infrared thermal camera. The experiment temperature was maintained at 25°C, and the environment humidity was between 30% and 35% during tests. The water evaporation rates of different experiments can be calculated by Eq. (12):

$E.R.=\frac{\Delta M}{At}$ (12)

Where E.R. (kg/m^2^/h) is the water evaporation rate per unit area, ∆M (kg) is the mass of water evaporated during the evaporation stage, A (m^2^) is the area of the GSPCAA, and t (h) represents the evaporation time.

The photothermal evaporation efficiency (η) was calculated by Eq. (13):

$\eta=\frac{E.R.(Lv+Q)}{C_{opt}xP}X$100% (13)

Where Lv refers to latent heat of phase change (it's usually taking 2260 kJ/kg), C_opt_ is the optical concentration (the number of the sun), P is the nominal direct illumination density (1 kW/m^2^) under 1 sun. Q is liquid-vapor phase change sensible heat which is calculated by Eq. (14):

$Q=C\Delta T$ (14)

Where C is specific heat capacity of water (usually refer to 4.2 kJ/kg/K), and ∆T refers to the temperature increment of the water.

**1.9.17 Dipole moment calculation**

All calculations were carried out with the Gaussian 16 software. The B3LYP functional was adopted for all calculations. For geometry optimization calculations, the def2-svp basis set was used. The DFT-D3 dispersion correction with BJ-damping was applied to correct the weak interaction to improve the calculation accuracy [25, 26].

**1.9.18 Density Functional Theory (DFT) Calculations**

The theoretical calculations were carried out within the density functional theory (DFT) framework to explore the H-bonds between chemical molecules including the GO, SL and PVA chain. The structural optimizations were performed by the DMol_3_ module on the Materials Studio software. The DFT Semi-core Pseudopots (DSPP) was employed to treat the core electrons, and the exchange-correlation effects were treated using the functional of Perdew-Burke-Ernzerhof (PBE) under the generalized gradient approximation (GGA). Meanwhile, the physical wave functions were expanded by the GGA-PBE/DNP (3.5) basis set. PVA, GO and SA chain was built. The geometrical configurations of the considered structures were visualized using the VESTA package. The binding energy of these structures including the PVA/PVA, PVA/GO, PVA/SL and SL/GO was determined. The binding energy indicates the interaction intensity between different chemical molecules. The more negative binding energy suggests the stronger interactions between different components [27].

**1.10 Statistical analysis**

Statistical analysis was performed on the data, which was presented as the standard deviation (SD) ± mean value. Error bars in the data plots indicated the SD calculated from at least five tests conducted on each sample. Data processing was carried out using Origin 2021. If not noted otherwise, each experiment was repeated for five times. If not noted otherwise, each experiment was repeated for five times.

**2. Results and discussion**





**Figure S1** Zeta potential of GSPCAA.





**Figure S2** XRD patterns of GO and SL.





**Figure S3** TGA curves of GO and SL.





**Figure S4** TGA curves of PPA, GPA, SPA and GSPCAA.


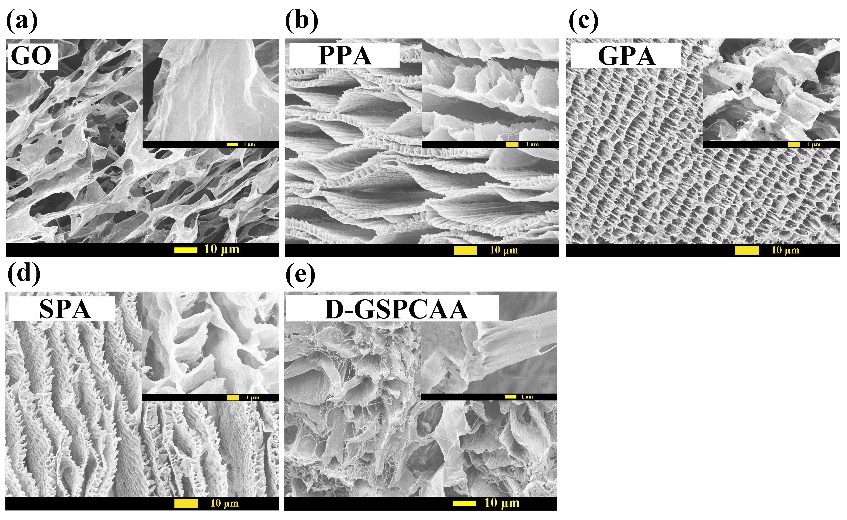


**Figure S5** SEM images of GO, PPA, GPA, SPA and D-GSPCAA.


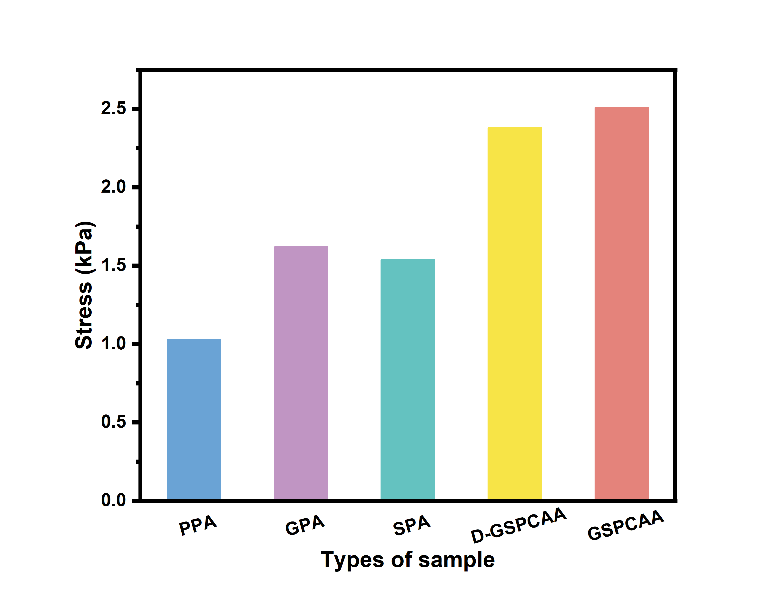


**Figure S6** Compressive stress of PPA, GPA, SPA, D-GSPCAA and GSPCAA at 70% strain amplitude.





**Figure S7** Solar energy absorption spectra of GO and SL.


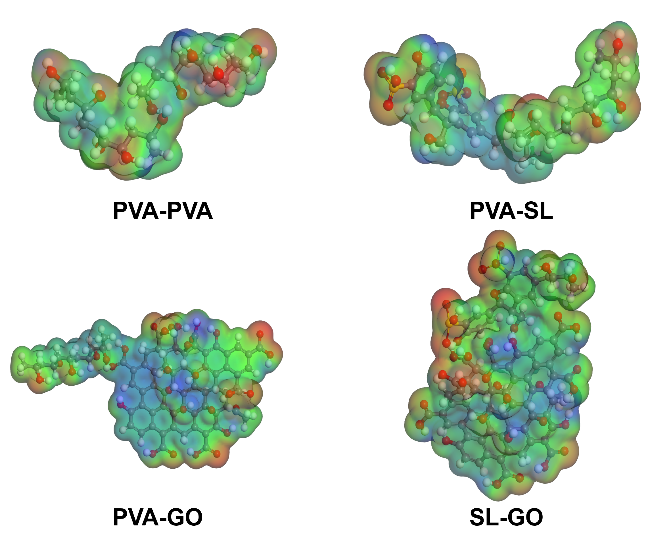


**Figure S8** The electrostatic potential distribution of the four pairs of components in the GSPCAA based on the DFT optimized structures.


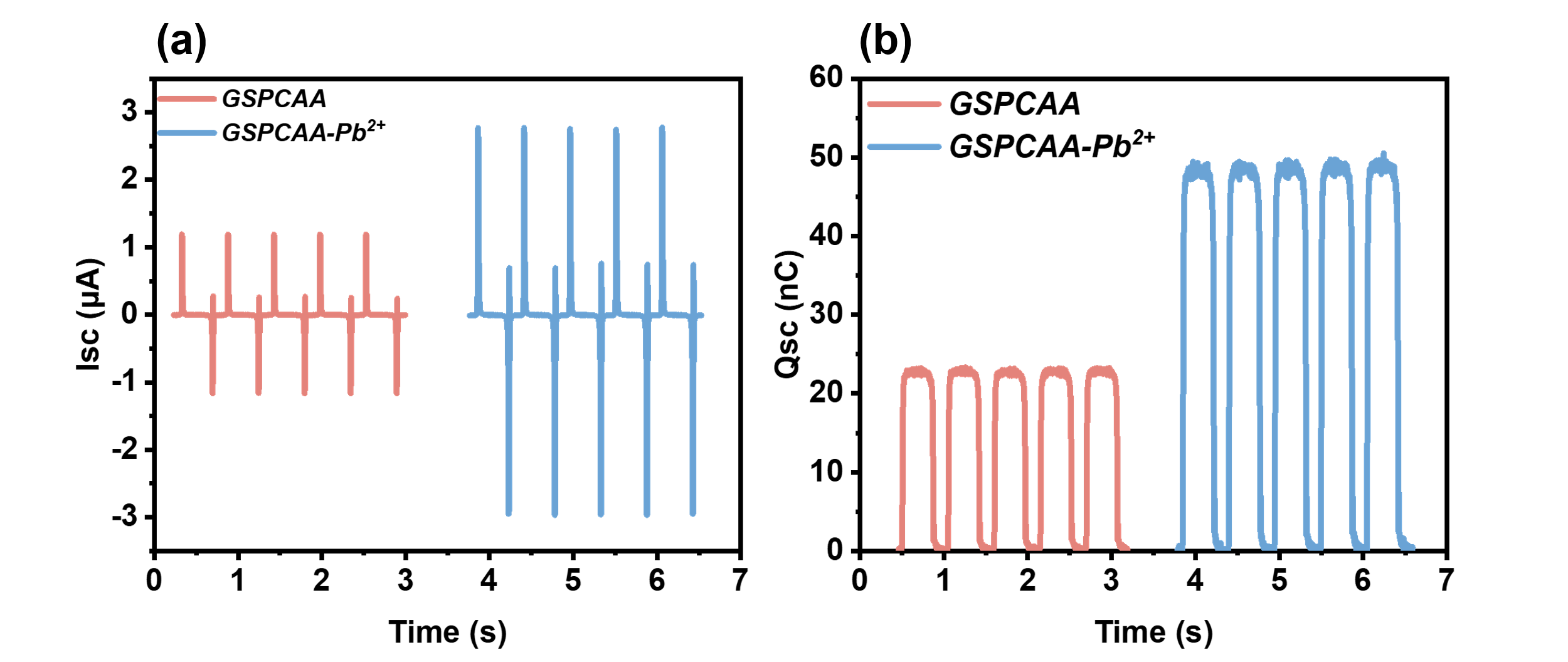


**Figure S9** Comparison of (a) Isc, and (b) Qsc of GSPCAA and GSPCAA-Pb^2+^.


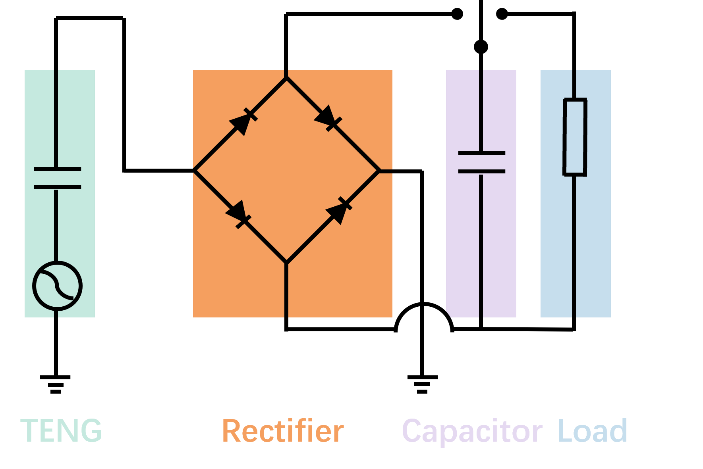


**Figure S10** Circuit diagram of triboelectric nanogenerator self-charging power system.

**Table S1** Adsorption isotherm model parameters of GSPCAA for Pb^2+^.

| **Condition** | **Langmuir model** | | | **Freundlich model** | | |
| --- | --- | --- | --- | --- | --- | --- |
|  | **K_L_ (L/g)** | **Q_max_ (mg/g)** | **R^2^** | **K_F_ (L/g)** | **1/n** | **R^2^** |
| Dark | 0.0958 | 246.9 | 0.9831 | 33.5984 | 0.0609 | 0.9231 |
| Light | 0.0506 | 315.8 | 0.9924 | 54.0863 | 0.0462 | 0.9472 |

**Table S2** Comparison of Pb^2+^ adsorption capacities of GSPCAA with other reported biomass-based adsorbents.

| **Adsorbent** | **Q_max_ (mg/g)** | **Adsorption condition** | **References** |
| --- | --- | --- | --- |
| **GSPCAA** | 315.8 | 328 K, 200 mg/L, pH=5, 48 h | This work |
| LMM | 187.4 | 303 K, 30 mg/L, pH = 5, 180 min | [28] |
| Carboxymethylated lignin | 107.5 | 30°C, 0.1 mol/dm^3,^ pH=6, 30 min | [29] |
| Soda lignin from oil palm | 46.1 | 47°C, 1000 mg/L, pH=6, 360 min | [30] |
| Crown functionalized lignin | 91.4 | 25°C, 200 mg/L, pH=6, 300 min | [31] |
| PEI functionalized lignin | 33.9 | 25°C, 50 mg/L, pH=6, 180 min | [32] |
| DLGS | 275.9 | 298K, 800 mg/L, pH=5.5, 180 min | [33] |
| MAL | 79.1 | 25°C, 30 mg/L, pH=6, 170 min | [34] |
| FLAL | 66.6 | 25°C, 100 mg/L, pH=6, 100 min | [35] |

**Table S3** Adsorption kinetic model parameters of GSPCAA for Pb^2+^.

| **Condition** | **Pseudo-first-order** | | | **Pseudo-second-order** | | |
| --- | --- | --- | --- | --- | --- | --- |
|  | **K_1_ (1/h)** | **Q_e_ (mg/g)** | **R^2^** | **K_2_ (g/mg/h)** | **Q_e_ (mg/g)** | **R^2^** |
| Dark | 0.0691 | 212.36 | 0.9877 | 0.1394 | 226.65 | 0.9913 |
| Light | 0.0743 | 213.19 | 0.9874 | 0.1736 | 225.71 | 0.9916 |

**Table S4** Adsorption isotherm model parameters of GSPCAA for Pb^2+^ at different temperature.

| **Temperature** | **Langmuir model** | | | **Freundlich model** | | |
| --- | --- | --- | --- | --- | --- | --- |
|  | **K_L_ (L/g)** | **Q_max_ (mg/g)** | **R^2^** | **K_F_ (L/g)** | **1/n** | **R^2^** |
| 298 K | 0.0406 | 256.7 | 0.9751 | 39.5772 | 0.0318 | 0.9231 |
| 308 K | 0.0461 | 278.2 | 0.9802 | 44.5798 | 0.0375 | 0.9277 |
| 318 K | 0.0492 | 293.1 | 0.9895 | 49.2683 | 0.0398 | 0.9396 |
| 328 K | 0.0506 | 315.8 | 0.9924 | 54.0863 | 0.0462 | 0.9472 |

**Table S5** Thermodynamics model parameters of GSPCAA for Pb^2+^.

| **Condition** | **ΔH (J/mol)** | **ΔS (J/mol/K)** | **ΔG (kJ/mol)** | | | |
| --- | --- | --- | --- | --- | --- | --- |
|  |  |  | **298 K** | **308 K** | **318 K** | **328 K** |
| Light | 8.06 | 93.53 | -27.86 | -28.79 | -29.73 | -30.67 |

**Table S6** Adsorption affinity of seven metal ions.

| **Condition** | **Pb^2+^** | **Co^2+^** | **Mg^2+^** | **Cd^2+^** | **Cu^2+^** | **Zn^2+^** |
| --- | --- | --- | --- | --- | --- | --- |
| K_d_ | 3.95×10^5^ | 2.45×10^3^ | 2.32×10^3^ | 3.87×10^3^ | 9.23×10^2^ | 6.87×10^2^ |

**Table S7** Comparison of output performances of GSPCAA-Pb^2+^ with previously reported biomass-based triboelectric materials.

| **Sample** | **Output performances (Voc)** | **Reference** |
| --- | --- | --- |
| GSPCAA-Pb^2+^ | 101.3 | This work |
| ZnCl_2_/EG/PAA-HPC | 55.6 | [36] |
| Zwitterionic | 21.8 | [37] |
| PVA | 4.5 | [38] |
| BC-CS/PDMS-Cu | 45 | [39] |
| BTO-PVDF-GO | 2.5 | [40] |
| PDMS-AgNWs-CNT | 21 | [41] |

**Table S8** The detailed parameters for constructing CNN networks.

| **Layer type** | **Number of Neurons** | **Number of filters** | **Kernel/Pool size** | **Stride** | **Activation functions** | **Padding type** | **Value** |
| --- | --- | --- | --- | --- | --- | --- | --- |
| Conv1D-1 | / | 64 | 2 | 1 | 0.001 | Valid | / |
| MaxPooling | / | / | / | 1 | / | Valid | / |
| Conv1D-2 | / | 32 | 2 | 1 | 0.001 | Valid | / |
| Flatten | / | / | / | / | / | / | / |
| Dense | 128 | / | / | / | 0.001 | / | / |
| Dropout rate | / | / | / | / | / | / | 0.5 |
| Learning rate | / | / | / | / | / | / | 0.0005 |
| Batch size | / | / | / | / | / | / | 64 |
| Number of epochs | / | / | / | / | / | / | 300 |
| Train split | / | / | / | / | / | / | 64% |
| Validation split | / | / | / | / | / | / | 16% |
| Test split | / | / | / | / | / | / | 20% |

**3. Reference**

[1] J. Chen, B. Yao, C. Li, G. Shi, An improved Hummers method for eco-friendly synthesis of graphene oxide, Carbon 64 (2013) 225-229.

[2] M.J. Uddin, R.E. Ampiaw, W. Lee, Adsorptive removal of dyes from wastewater using a metal-organic framework: A review, Chemosphere 284 (2021) 131314.

[3] R. Nodehi, H. Shayesteh, A. Rahbar-Kelishami, Fe3O4@ NiO core–shell magnetic nanoparticle for highly efficient removal of Alizarin red S anionic dye, International Journal of Environmental Science and Technology 19(4) (2022) 2899-2912.

[4] C. Feng, P. Ren, M. Huo, Z. Dai, D. Liang, Y. Jin, F. Ren, Facile synthesis of trimethylammonium grafted cellulose foams with high capacity for selective adsorption of anionic dyes from water, Carbohydrate Polymers 241 (2020) 116369.

[5] E.H.C. de Oliveira, D.M.d.S.M. Fraga, M.P. da Silva, T.J.M. Fraga, M.N. Carvalho, E.M.P. de Luna Freire, M.G. Ghislandi, M.A. da Motta Sobrinho, Removal of toxic dyes from aqueous solution by adsorption onto highly recyclable xGnP® graphite nanoplatelets, Journal of Environmental Chemical Engineering 7(2) (2019) 103001.

[6] T. Cai, H. Li, R. Yang, Y. Wang, R. Li, H. Yang, A. Li, R. Cheng, Efficient flocculation of an anionic dye from aqueous solutions using a cellulose-based flocculant, Cellulose 22(2) (2015) 1439-1449.

[7] M. Kavand, P. Eslami, L. Razeh, The adsorption of cadmium and lead ions from the synthesis wastewater with the activated carbon: Optimization of the single and binary systems, Journal of Water Process Engineering 34 (2020) 101151.

[8] J.-L. Wen, T.-Q. Yuan, S.-L. Sun, F. Xu, R.-C. Sun, Understanding the chemical transformations of lignin during ionic liquid pretreatment, Green Chem. 16(1) (2014) 181-190.

[9] J.-L. Wen, S.-L. Sun, T.-Q. Yuan, F. Xu, R.-C. Sun, Understanding the chemical and structural transformations of lignin macromolecule during torrefaction, Applied Energy 121 (2014) 1-9.

[10] L.-P. Xiao, Y.-Y. Bai, Z.-J. Shi, Q. Lu, R.-C. Sun, Influence of alkaline hydrothermal pretreatment on shrub wood Tamarix ramosissima : Characteristics of degraded lignin, Biomass and Bioenergy 68 (2014) 82-94.

[11] J.-L. Wen, B.-L. Xue, F. Xu, R.-C. Sun, A. Pinkert, Unmasking the structural features and property of lignin from bamboo, Industrial Crops and Products 42 (2013) 332-343.

[12] Z. Li, J. Chen, Y. Ge, Removal of lead ion and oil droplet from aqueous solution by lignin-grafted carbon nanotubes, Chemical Engineering Journal 308 (2017) 809-817.

[13] T.V. Charpentier, A. Neville, J.L. Lanigan, R. Barker, M.J. Smith, T. Richardson, Preparation of magnetic carboxymethylchitosan nanoparticles for adsorption of heavy metal ions, ACS omega 1(1) (2016) 77-83.

[14] A. Tzereme, E. Christodoulou, G.Z. Kyzas, M. Kostoglou, D.N. Bikiaris, D.A. Lambropoulou, Chitosan grafted adsorbents for diclofenac pharmaceutical compound removal from single-component aqueous solutions and mixtures, Polymers 11(3) (2019) 497.

[15] Y. Zhang, S. Ni, X. Wang, W. Zhang, L. Lagerquist, M. Qin, S. Willför, C. Xu, P. Fatehi, Ultrafast adsorption of heavy metal ions onto functionalized lignin-based hybrid magnetic nanoparticles, Chemical Engineering Journal 372 (2019) 82-91.

[16] M. Ma, L. Dai, J. Xu, Z. Liu, Y. Ni, A simple and effective approach to fabricate lignin nanoparticles with tunable sizes based on lignin fractionation, Green Chemistry 22(6) (2020) 2011-2017.

[17] M. Zhu, H. Liu, Q. Cao, H. Zheng, D. Xu, H. Guo, S. Wang, Y. Li, J. Zhou, Electrospun Lignin-Based Carbon Nanofibers as Supercapacitor Electrodes, ACS Sustainable Chemistry & Engineering 8(34) (2020) 12831-12841.

[18] B. Liu, B. Du, Y. Sun, M. Zhu, Y. Yang, X. Wang, J. Zhou, Ultrasound acoustic cavitation enhances depolymerization of organosolv lignin to phenolic monomers and low molecular weight lignin bio-oils, Fuel Processing Technology 203 (2020).

[19] T. Zhao, J. Wang, Y. Liu, X. Li, Y. Bai, B. Luo, M. Chi, S. Zhang, T. Liu, Y. Shao, Self‐Healing and Toughness Triboelectric Materials Enabled by Dynamic Nanoconfinement Quenching, Advanced Functional Materials 34(51) (2024) 2410096.

[20] B. Du, W. Li, H. Zhu, J. Xu, Q. Wang, X. Shou, X. Wang, J. Zhou, A functional lignin for heavy metal ions adsorption and wound care dressing, International Journal of Biological Macromolecules 239 (2023) 124268.

[21] Y. Wang, H. Zhang, Influence of asphalt microstructure to its high and low temperature performance based on atomic force microscope (AFM), Construction and Building Materials 267 (2021) 120998.

[22] Y. Gao, P.-H. Yu, J. Zhang, G.-D. Zhang, C.-H. Guo, Y.-Q. Zhou, Y.-Z. Long, H. Wu, Compressible Piezoelectric Ceramic Nanofiber Aerogels with Multifunction, Advanced Fiber Materials 7(3) (2025) 937-949.

[23] M. Fu, L. Han, C. Huang, L. Ma, Y. Yao, K. Chen, P. Sun, H. Shao, M. Wu, B. Zhang, Janus photothermal adsorbent for solar-powered simultaneous uranium extraction and co-production of freshwater and sea salt from seawater, Chemical Engineering Journal 477 (2023) 147103.

[24] G.-J. Jiao, J. Ma, J. Hu, X. Wang, R. Sun, Hierarchical build-up of vertically oriented lignin-based aerogel for photothermally assisted uranium uptake and recovery from acidic wastewater, Journal of Hazardous Materials 448 (2023) 130988.

[25] J. Wang, Y. Chen, Y. Xu, J. Mu, J. Li, S. Nie, S. Chen, F. Xu, Sustainable lignin-based electrospun nanofibers for enhanced triboelectric nanogenerators, Sustainable Energy & Fuels 6(8) (2022) 1974-1982.

[26] C. Li, J. Yue, B. Wang, Y. Tao, J. Hu, J. Lu, J. Du, H. Wang, Unveiling the mechanism of lignin nanofiller in boosting the performance of triboelectric nanogenerators, Nano Energy 126 (2024) 109631.

[27] Z. Sun, C. Dang, H. Zhang, Y. Feng, M. Jiang, S. Hu, Y. Shao, S. Hao, C. Shao, W. Zhai, Lignin powered versatile bioelastomer: a universal medium for smart photothermal conversion, Advanced Functional Materials 34(45) (2024) 2405130.

[28] Z. Zhang, Y. Chen, C. Wu, Dual-Modified Lignin-Assembled Multilayer Microsphere with Excellent Pb2+ Capture, Polymers 14(14) (2022) 2824.

[29] W.S. Peternele, A.A. Winkler-Hechenleitner, E.A.G. Pineda, Adsorption of Cd (II) and Pb (II) onto functionalized formic lignin from sugar cane bagasse, Bioresource Technology 68(1) (1999) 95-100.

[30] M.M. Ibrahim, W.W. Ngah, M. Norliyana, W.W. Daud, M. Rafatullah, O. Sulaiman, R. Hashim, A novel agricultural waste adsorbent for the removal of lead (II) ions from aqueous solutions, Journal of hazardous materials 182(1-3) (2010) 377-385.

[31] C. Jin, G. Liu, G. Wu, S. Huo, Z. Liu, Z. Kong, Facile fabrication of crown ether functionalized lignin-based biosorbent for the selective removal of Pb (II), Industrial Crops and Products 155 (2020) 112829.

[32] Y. Ge, L. Qin, Z. Li, Lignin microspheres: An effective and recyclable natural polymer-based adsorbent for lead ion removal, Materials & Design 95 (2016) 141-147.

[33] X. Lin, T. Shen, M. Li, J. Shaoyu, W. Zhuang, M. Li, H. Xu, C. Zhu, H. Ying, P. Ouyang, Synthesis, characterization, and utilization of poly-amino acid-functionalized lignin for efficient and selective removal of lead ion from aqueous solution, Journal of Cleaner Production 347 (2022) 131219.

[34] Q. Wang, C. Zheng, Z. Shen, Q. Lu, C. He, T.C. Zhang, J. Liu, Polyethyleneimine and carbon disulfide co-modified alkaline lignin for removal of Pb2+ ions from water, Chemical Engineering Journal 359 (2019) 265-274.

[35] Q. Wang, C. Zheng, W. Cui, F. He, J. Zhang, T.C. Zhang, C. He, Adsorption of Pb2+ and Cu2+ ions on the CS2-modified alkaline lignin, Chemical Engineering Journal 391 (2020) 123581.

[36] C. Lu, X. Wang, Y. Shen, S. Xu, C. Huang, C. Wang, H. Xie, J. Wang, Q. Yong, F. Chu, Skin‐Like Transparent, High Resilience, Low Hysteresis, Fatigue‐Resistant Cellulose‐Based Eutectogel for Self‐Powered E‐Skin and Human–Machine Interaction, Advanced Functional Materials 34(13) (2024) 2311502.

[37] J. Yang, L. Chang, H. Deng, Z. Cao, Zwitterionic eutectogels with high ionic conductivity for environmentally tolerant and self-healing triboelectric nanogenerators, ACS nano 18(29) (2024) 18980-18991.

[38] S. Du, X. Chen, M. Li, B. Peng, Q. Lyu, L. Zhang, J. Zhu, Ultratough and Highly Conductive Supramolecular Poly (Vinyl Alcohol) Eutectogels via a Sequentially Enhanced Strategy, Advanced Functional Materials 35(2) (2025) 2409726.

[39] J. Huang, Y. Hao, M. Zhao, H. Qiao, F. Huang, D. Li, Q. Wei, Biomass-based wearable and Self-powered pressure sensor for human motion detection, Composites Part A: Applied Science and Manufacturing 146 (2021) 106412.

[40] M. Zhu, M. Lou, I. Abdalla, J. Yu, Z. Li, B. Ding, Highly shape adaptive fiber based electronic skin for sensitive joint motion monitoring and tactile sensing, Nano Energy 69 (2020) 104429.

[41] C. Ning, K. Dong, R. Cheng, J. Yi, C. Ye, X. Peng, F. Sheng, Y. Jiang, Z.L. Wang, Flexible and stretchable fiber‐shaped triboelectric nanogenerators for biomechanical monitoring and human‐interactive sensing, Advanced Functional Materials 31(4) (2021) 2006679.
